# Synergistic Optimization of In Vitro Digestibility and Sensory Quality of Moderately Milled Rice Based on the RiceMambaOpt Model

**DOI:** 10.3390/foods15162812

**Published:** 2026-08-12

**Authors:** Zijun Li, Zhihong Wen, Mengting Ma, Wenshu Niu, Zhongquan Sui, Harold Corke

**Affiliations:** 1 Department of Food Science & Technology, School of Agriculture and Biology, Shanghai Jiao Tong University, Shanghai 200240, China; zijunli@sjtu.edu.cn (Z.L.); mmt9497@sjtu.edu.cn (M.M.); 2 College of Information, Mechanical and Electrical Engineering, Shanghai Normal University, Shanghai 201418, China; 1000555469@smail.shnu.edu.cn (Z.W.); 1000573960@smail.shnu.edu.cn (W.N.); 3 Biotechnology and Food Engineering Program, Guangdong Technion-Israel Institute of Technology, Shantou 515063, China; harold.corke@gtiit.edu.cn; 4 Faculty of Biotechnology and Food Engineering, Technion-Israel Institute of Technology, Haifa 3200003, Israel

**Keywords:** rice processing, artificial intelligence, deep learning, inverse design, intelligent control, moderate processing

## Abstract

To address over-processing and limited process-control precision in rice manufacturing, this study developed an artificial intelligence (AI)-assisted optimization framework for rice milling. A data-driven predictive model was constructed to characterize the nonlinear relationships between milling conditions and the in vitro starch-digestibility and sensory attributes of rice. Across the nine physicochemical, in vitro starch-digestibility, and sensory indicators, RiceMambaOpt achieved a mean coefficient of determination (*R*^2^) of 0.975. Explainability analyses were used to examine process–quality relationships and characterize nonlinear trade-offs among appearance, texture, and starch-digestibility attributes across rice cultivars with different genetic backgrounds. A target-oriented inverse optimization procedure was then developed, which estimates feasible process parameters subject to process-feasibility constraints, tailored to differentiated orientations such as low rapidly digestible starch (RDS) content or high palatability. On the independent 100-sample test set, the inverse predictions achieved a milling-time MAE of 0.960 s with an *R*^2^ of 0.986 and a milling-speed MAE of 19.522 r/min with an *R*^2^ of 0.851. In a prospective experimental validation, 10 of the 12 prespecified target quality profiles (83.3%) were attained across 36 independently milled samples, with an RDS mean absolute error of 1.8 percentage points and a joint normalized root-mean-square error of 6.7%. For the Qiuguang cultivar, the model identified a representative processing condition of 38.5 s and 1020 r/min, corresponding to a predicted in vitro RDS content of 21.6% and a palatability score of 26.5. The results demonstrate the feasibility of combining predictive modeling with process-feasibility-constrained inverse optimization and provide a computational approach for investigating moderate rice-milling conditions.

## 1. Introduction

Rice is the staple food for more than half of the world’s population, and its processing quality and nutritional properties are directly associated with dietary health outcomes [1]. For decades, in pursuit of a whiter appearance and a softer texture, the rice processing industry has widely adopted excessive milling practices [2,3]. This not only causes substantial losses of micronutrients in the bran layer, including dietary fiber, vitamins, and minerals [4], but also markedly alters starch digestibility, making polished rice a high-glycemic-index (GI) food and thereby increasing the risk of metabolic disorders such as obesity and type 2 diabetes [5]. Recent process-level investigations have further quantified the practical consequences of overmilling. For round-grain late-*japonica* rice, moderate milling reduced endosperm loss by 1.65–2.76 percentage points, embryo loss by 27.03–34.80 percentage points, broken-rice production by 3.67–17.84 percentage points, and unit energy consumption by 1.20–12.60 kJ/kg compared with well-milled rice [6]. Machine-learning-based prediction of the degree of milling has also been investigated as a means of supporting real-time identification of overmilling and intelligent process adjustment [7]. Against this backdrop, developing the whole-grain industry and promoting moderate grain-processing technologies serve as critical measures to address these issues. However, in practical production, moderate processing often faces a fundamental trade-off between eating quality and nutritional value [8]: retaining more of the bran layer can increase the contents of slowly digestible starch (SDS) and resistant starch (RS) while reducing the proportion of rapidly digestible starch (RDS), but it is often accompanied by undesirable sensory changes, such as increased hardness, reduced viscosity, and poorer palatability. Therefore, a key scientific challenge in rice processing is how to precisely regulate milling parameters to maximize nutritional retention while maintaining sensory quality at a level acceptable to consumers, thereby identifying the optimal balance in processing intensity [9].

At present, optimization studies on rice milling are still mainly based on response surface methodology and orthogonal experimental design [10]. Although these conventional approaches perform well in handling single-factor effects or low-dimensional linear relationships, they often fail to identify the global optimum when confronted with the highly nonlinear coupling between multiple processing parameters and multidimensional quality attributes during rice milling. In recent years, machine learning has gradually been introduced into food processing prediction, detection, and quality-control tasks [11,12], and broader AI applications are increasingly being explored across food production and sensory science [13]. However, rice milling experiments are constrained by raw material batches, analytical costs, and labor requirements, making it difficult to obtain large volumes of labeled data. Under small-sample conditions, deep learning models are highly susceptible to overfitting [14,15]. Moreover, deep learning is not universally superior for structured tabular data. Controlled benchmarking studies have demonstrated that gradient-boosted trees and random forests frequently remain competitive with, or outperform, deep architectures on small- and medium-sized tabular datasets because they are comparatively robust to uninformative features, heterogeneous feature scales, and limited sample sizes [16,17,18]. CatBoost and XGBoost are therefore particularly relevant baselines for the present dataset, while Gaussian process regression offers an additional sample-efficient probabilistic alternative with explicit uncertainty estimation. Consequently, the suitability of a complex neural architecture for the current application must be demonstrated through matched empirical comparison rather than assumed from its architectural novelty. In addition, most existing data-driven models function as black-box mappings, offering limited mechanistic interpretation of the physical milling process [19,20]. During inverse process design, they often focus solely on minimizing numerical error while overlooking practical physical constraints. This can lead to ineffective parameter recommendations, such as extremely low milling speeds or excessively short milling times, which fail to remove the bran layer in real-world processing.

**Figure 1:**
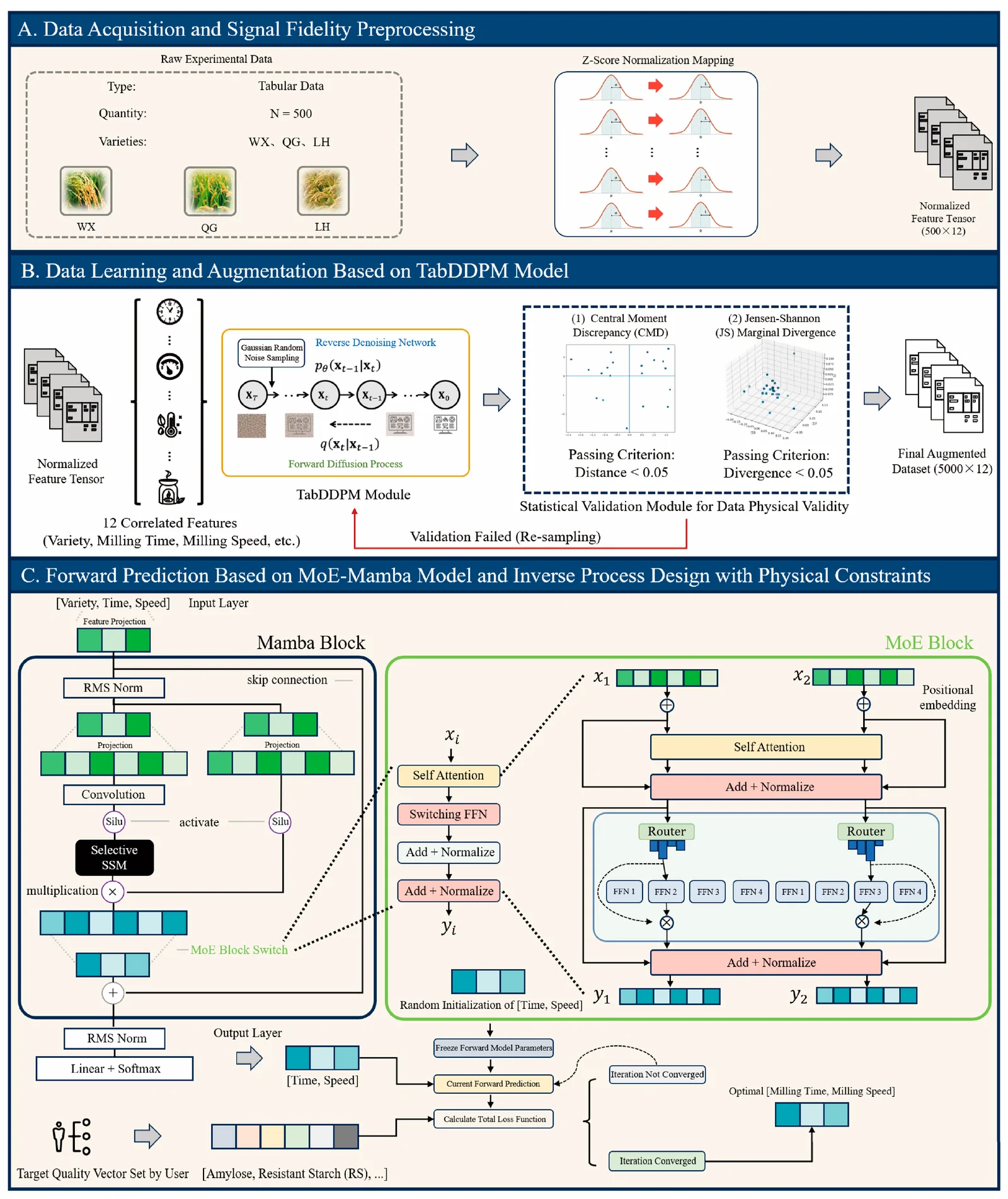
Overall workflow of the RiceMambaOpt system, including experimental data acquisition and standardization, leakage-controlled TabDDPM augmentation, MoE–Mamba forward prediction, process-feasibility-constrained inverse optimization, and independent validation and interpretability analysis. Arrows indicate data or optimization flow, and colors distinguish functional modules and feature groups.

To address these challenges, this study proposes RiceMambaOpt, an inverse optimization framework for moderate rice processing that integrates process-feasibility constraints with generative artificial intelligence (see Figure 1). First, to overcome data scarcity, a data augmentation strategy based on a tabular diffusion probabilistic model (TabDDPM) was introduced to expand the process sample space while preserving the physical distribution characteristics of the original data. Second, as rice milling is not an instantaneous mapping but rather a dynamic physical process in which the effects of milling time and rotational speed accumulate during progressive removal of the grain surface layers, a state-space model (Mamba) was adopted as the forward predictor to capture the cumulative temporal effects of processing parameters [21,22]. This predictor was further combined with a Mixture of Experts (MoE) architecture to adaptively learn the distinct fracture behaviors and quality response mechanisms of *indica*, *japonica*, and glutinous rice under different processing intensities. Finally, during the inverse optimization stage, a loss function incorporating a normalized process-dose feasibility penalty and equipment operating limits was constructed, and the forward model was treated as a differentiable operator to solve for the optimal milling parameters in a continuous parameter space. In this way, the resulting solutions simultaneously satisfy multiple objectives, including enhanced slow digestibility, high palatability, and engineering feasibility. This study aims to provide both a theoretical basis and technical support for the standardized, intelligent, and precision processing of whole-grain rice products and of rice products with a reduced in vitro RDS fraction. The adoption of RiceMambaOpt was therefore motivated primarily by the need for a differentiable forward operator that supports gradient-based process inversion, cultivar-adaptive routing, and joint uncertainty estimation. Its predictive performance was not presumed to be superior solely because it is a deep architecture; instead, it was evaluated against well-tuned quadratic RSM, SVR, RF, XGBoost, GPR, CatBoost, and MLP models under identical leakage-controlled data partitions and comparable hyperparameter-selection procedures.

## 2. Materials and Methods

### 2.1. Experimental Materials and Data Acquisition

Three representative rice cultivars were selected in this study: Yangfunuo 4, a glutinous rice cultivar with an extremely low amylose content (approximately 1.58%); Qiuguang, a *japonica* rice cultivar with a moderate amylose content (approximately 16.68%); and Luhui 6684, an *indica* rice cultivar with a relatively high amylose content (approximately 23.1%). These cultivars were chosen to capture differences in rice responses to milling-induced mechanical forces. All paddy samples were dehulled using a rice husker (JLG-II, Zhongchuliang Corporation, Chengdu, China) to obtain brown rice, and immature and broken kernels were manually removed to ensure the consistency of the experimental materials. Before milling, the initial moisture content, thousand-kernel weight, kernel length and width, length-to-width ratio, single-kernel compression hardness, and amylose content of each cultivar were determined. Moisture content and thousand-kernel weight were measured in three independent batches, whereas kernel dimensions and compression hardness were measured using 30 randomly selected intact kernels from each cultivar. The initial physical characteristics are reported in Appendix A—Table A1.

Milling treatment was conducted using a laboratory-scale precision rice mill (TM 05C, Satake Corporation, Tokyo, Japan). To construct a continuous processing space covering the full range from under-milling to over-milling, milling time (X_1_) and milling speed (X_2_) were selected as the key input variables. The experimental design combined a full-factorial scheme with random sampling. Milling time ranged from 0 to 60 s, and milling speed ranged from 0 to 1050 r/min. This parameter range effectively simulated the entire processing continuum, from slight surface abrasion of brown rice to complete bran removal and deep milling of polished rice. A total of 500 valid samples were prepared and collected, including 177 samples of Yangfunuo 4, 155 samples of Qiuguang, and 168 samples of Luhui 6684, thereby ensuring an even distribution of each cultivar across the processing space and adequate data representativeness. For every milling run, the brown-rice sample mass was fixed at 100.0 ± 0.1 g, and the outlet-resistance counterweight was maintained at 1.0 kg, corresponding to a nominal axial load of 9.81 N. The counterweight position and milling-chamber geometry were unchanged throughout the experiment. Since the sample mass and nominal load were held constant and therefore contained no between-sample variation, they were controlled experimental conditions rather than model input variables. The actual transient grain-contact pressure was not measured; consequently, no unsupported pressure value in kPa was assigned.

For each experimental sample, starch digestibility was systematically evaluated using an in vitro simulated digestion method [23]. The percentages of rapidly digestible starch (RDS), slowly digestible starch (SDS), and resistant starch (RS) were quantified and used as key indicators for assessing the glycemic index (GI) potential of rice, which is strongly associated with cultivar-specific starch physicochemical properties [24]. Amylose content was determined by the iodine colorimetric method [25] to characterize the genetic and physicochemical basis of each cultivar. The sensory evaluation was conducted strictly following the Chinese national standard GB/T 15682-2025 [26]. Ten panelists (five males and five females aged 20–30 years) recruited from Shanghai Jiao Tong University performed the test, and all panelists received standardized training for rice sensory evaluation as required by the standard. Rice samples were prepared in line with the standard procedures, and freshly cooked rice was loaded into small bowls on white plates for sensory assessment. No raw or uncooked rice kernels were presented to the panelists. The unmilled brown-rice control and lightly milled samples were cooked according to the same standardized procedure and evaluated using the same sensory protocol as the moderately and deeply milled samples. Five attributes of cooked rice were evaluated: aroma, appearance, palatability, taste, and texture of cooled rice. Each rice sample was prepared in three independent cooking replicates, and every replicate was evaluated by all 10 trained panelists, yielding 30 ratings per sample. All samples were identified using random three-digit codes, and panelists were blinded to cultivar identity, milling time, milling speed, and model recommendation. Sample-presentation order was balanced across panelists and assessment sessions using a Williams Latin-square design. No more than six samples were evaluated in a single session. A minimum 10 min break was provided between sample sets, and water and unsalted crackers were used for palate cleansing. Ten percent of the samples were presented as blind duplicates to evaluate within-panelist repeatability. Inter-panelist agreement was assessed separately for each attribute using a two-way random-effects, absolute-agreement intraclass correlation coefficient for the panel mean, ICC (2,k), and Kendall’s coefficient of concordance (W). Repeatability across the three independent cooking replicates was evaluated using the coefficient of variation. These measurements were interpreted as trained-panel analytical sensory scores and not as instrumental measurements or consumer hedonic responses. The complete agreement and repeatability results are reported in Appendix A—Table A2.

After data cleaning and integration, an original dataset containing 500 samples was established. Statistical analysis of the dataset showed marked differences in quality responses among cultivars under the same processing range (Table 1). Under high-intensity milling conditions, Luhui 6684 exhibited a notably high resistant starch content, whereas the RDS content of Yangfunuo 4 remained consistently high. These findings indicate that the genetic background of each cultivar constrains the physical boundaries of process optimization and also provide data support for the subsequent construction of a cultivar-specific Mixture of Experts (MoE) model.

### 2.2. Data Standardization

**Figure 2:**
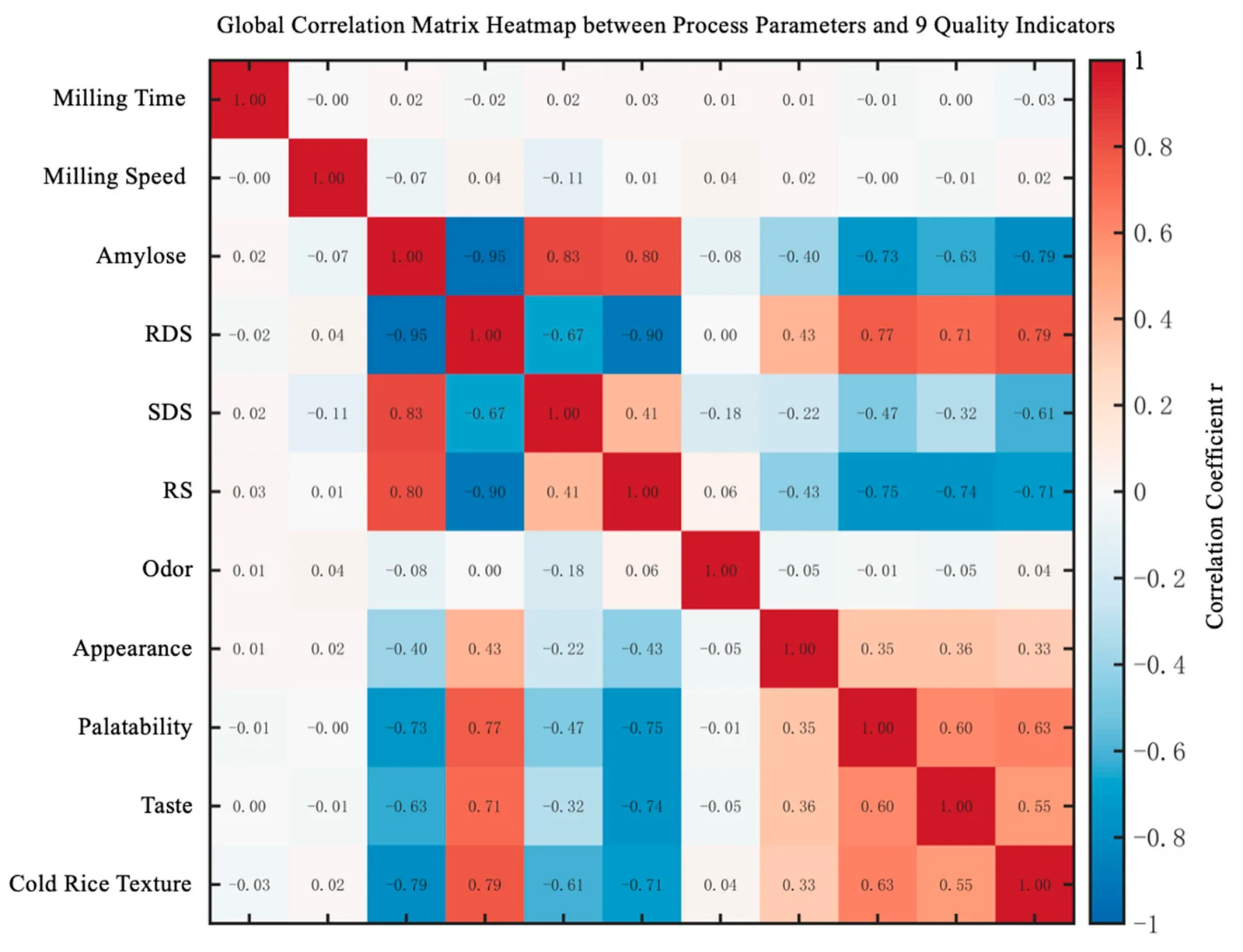
Pearson correlation heatmap.

In this study, the nine selected key quality indicators covered both physicochemical composition and sensory scores, and substantial differences in units and numerical scales existed among them. If the raw data were directly input into the deep learning model, variables with larger numerical values would dominate the direction of gradient descent, causing the model to be less sensitive to variables with smaller magnitudes and thereby reducing overall predictive accuracy. To address this issue, the raw data were first subjected to Z-score standardization. For each feature x in the dataset, the value was transformed to a standard normal distribution with a mean of 0 and a variance of 1 according to the following equation: Z-score standardization was the only numerical signal-preprocessing transformation used in this study. No discrete or continuous wavelet transform, wavelet denoising, wavelet decomposition, or wavelet-based feature extraction was performed.

(1) $$z=\frac{\left(x-\mu \right)}{\sigma }$$

where μ is the sample mean and σ is the standard deviation. This transformation eliminated scale-related bias and accelerated model convergence. Subsequently, to investigate the coupling relationships between the input processing parameters and the output quality attributes, Pearson correlation coefficients were calculated, and a correlation heatmap was generated (see Figure 2).

### 2.3. Small-Sample Data Augmentation Based on a Tabular Diffusion Model

Although 500 high-quality samples were obtained through a full-factorial experiment, this sample size remained relatively sparse within the high-dimensional feature space defined by cultivar, processing conditions, and quality attributes for constructing a deep neural network with complex nonlinear mappings. To improve model generalization without incurring additional experimental costs, a tabular diffusion probabilistic model (TabDDPM) was used to augment the original dataset. TabDDPM is a generative model [27] whose core framework consists of a forward diffusion process and a reverse denoising process. In the forward process, Gaussian noise is gradually added to the real data through a Markov chain until it degenerates into pure noise. In the reverse process, a denoising neural network is trained to predict and remove the noise in reverse order, thereby learning the underlying joint probability distribution of the data. Recent mixed-type tabular diffusion models further support the use of diffusion-based synthesis for preserving heterogeneous feature distributions and inter-column dependence in small, structured datasets [28,29]. Before any standardization, augmentation, or model fitting, the 500 real samples were randomly partitioned using a fixed seed of 2024 into a development set containing 400 samples and an untouched independent holdout set containing 100 samples. Five-fold cross-validation was performed only within the 400-sample development set. Each fold therefore contained 320 real training samples and 80 real validation samples. The standardization statistics and TabDDPM parameters were fitted separately using only the 320 real training samples in the corresponding fold. Synthetic samples were added exclusively to that fold’s training subset, whereas the validation and holdout subsets always contained real observations only. After selection of the synthetic-to-real ratio by cross-validation, the final model was fitted using the 400 real development samples and fold-safe synthetic augmentation and was evaluated once on the untouched 100-sample real holdout set.

**Figure 3:**
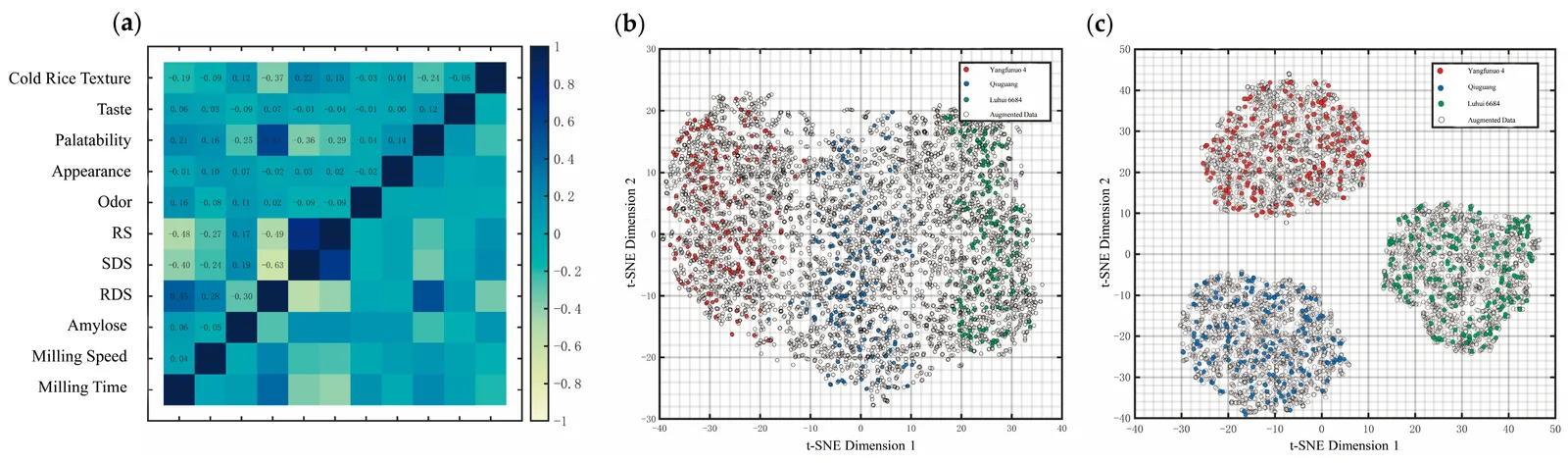
Consistency verification of feature distribution between real and augmented data. Colored points identify cultivars (real data), and open gray circles denote augmented observations. (**a**) Correlation matrix heatmap comparison; (**b**) continuous distribution structure across cultivars after weakening genetic characteristics; (**c**) cultivar-specific clustering in the full feature space. These analyses evaluate distributional similarity within the available experimental domain and should not be interpreted as evidence that the synthetic observations provide new physical information.

Using the converged TabDDPM model, 6500 candidate synthetic samples were randomly generated within the original process space. To prevent the generated data from violating basic physical processing principles, a physical-consistency screening strategy was introduced. Before calculation of dataset-level distribution metrics, every candidate sample was subjected sequentially to four plausibility checks. First, milling time and speed were required to remain within the equipment limits of 0–60 s and 0–1050 r/min, respectively, and the cultivar label was required to correspond to one of the three experimentally studied cultivars. Second, amylose, RDS, SDS, and RS values were required to be nonnegative; RDS + SDS + RS was required to remain within the training-derived analytical interval of 80–95%; and sensory attributes were required to remain within their prescribed scoring scales. Third, cultivar-dependent physicochemical variables were required to fall within the corresponding real-training envelope expanded by 5% of the interquartile range to accommodate analytical variability without permitting implausible extrapolation. Fourth, each candidate was compared with its five nearest real training samples of the same cultivar in standardized process space, and all generated quality attributes were required to remain within the local real-sample range expanded by two training-fold standard deviations. Specifically, the correlation matrix distance (CMD) between the real and generated datasets and the Jensen–Shannon (JS) divergence of their marginal distributions were calculated, following recent food-quality recommendations for multivariate data validation and digitalized food analysis [30,31]. Candidate generation and sample-level screening were continued until 4500 samples had been retained. The final retained set was accepted only when the dataset-level CMD was below 0.05 and the JS divergence for every modeled variable was below 0.05. For the descriptive distribution comparisons presented in Figure 3, the 4500 retained synthetic samples were combined with the 500 real samples to construct a 5000-sample visualization dataset; this globally generated dataset was not used for cross-validation or holdout evaluation. For predictive modeling, the entire generation and screening procedure was repeated independently within every training fold after the real observations had been partitioned into training and validation subsets, and all numerical limits and local-neighbor envelopes were calculated exclusively from that fold’s 320 real training samples. No real validation or holdout observation was used to define, screen, or verify a synthetic sample.

As shown in Figure 3a, the lower triangular matrix represents the correlation coefficients of the real data, whereas the upper triangular matrix represents those of the augmented data. The two matrices were highly symmetric in both color distribution and correlation structure, indicating that the augmented data showed strong consistency with the real data in preserving the underlying physical and statistical patterns, particularly the coupling relationships among variables. To further verify the distributional consistency between the augmented and real datasets, t-SNE visualization was performed at two levels: one emphasizing process- and sensory-dominated features and the other preserving genetically dominated features. The results demonstrated that, after weakening genetically deterministic features, samples from different cultivars exhibited partial overlap and a continuous distribution pattern in the two-dimensional embedding space, reflecting the regulatory effect of milling conditions on quality formation as well as similar cross-cultivar response patterns. In contrast, within the full feature space, the three rice cultivars formed well-defined cluster structures, and the augmented data were strictly distributed within their corresponding cultivar clusters, showing a high degree of overlap with the real data. These results indicate that rice quality data simultaneously possess continuous process-response characteristics and discrete cultivar-specific genetic structures. The TabDDPM model used in this study was able not only to preserve cultivar-specific distributions but also to capture continuous variation patterns across cultivars, thereby confirming the authenticity and physical plausibility of the augmented data. In other words, the generated data not only covered the numerical range of the real data but, more importantly, also accurately reproduced the correlation structure between processing parameters and quality attributes, demonstrating a high degree of physical credibility and providing sufficient data support for subsequent training of the MoE-Mamba model. Of the 6500 candidates generated for the descriptive augmented dataset, 2000 were rejected and 4500 were retained, corresponding to an overall retention rate of 69.2%. The retained set contained no equipment-bound, scoring-scale, starch-composition, cultivar-envelope, or local-support violations. Its final CMD was 0.018, and its maximum variable-wise JS divergence was 0.037. Across the five-fold-specific generators, the mean sample-retention rate was 69.2% ± 1.1%, indicating that the screening outcome was stable across training partitions. Detailed rejection counts and criteria are reported in Appendix A—Table A3.

### 2.4. Construction of the RiceMambaOpt Process Inversion System

The RiceMambaOpt system developed in this study was designed to establish a bidirectional mapping mechanism between rice milling parameters and multidimensional quality attributes. Since the milling process is characterized by pronounced nonlinearity and time-varying behavior, directly constructing an inverse regression model from quality attributes to process parameters is highly prone to nonconvergence due to the multiplicity of physical solutions; that is, different process combinations may yield similar quality outcomes. Therefore, a two-stage strategy was adopted in this study, in which forward physical modeling was first established and then used to drive inverse gradient-based optimization.

#### 2.4.1. Cultivar-Aware Forward Prediction Model Based on MoE-Mamba

**Figure 4:**
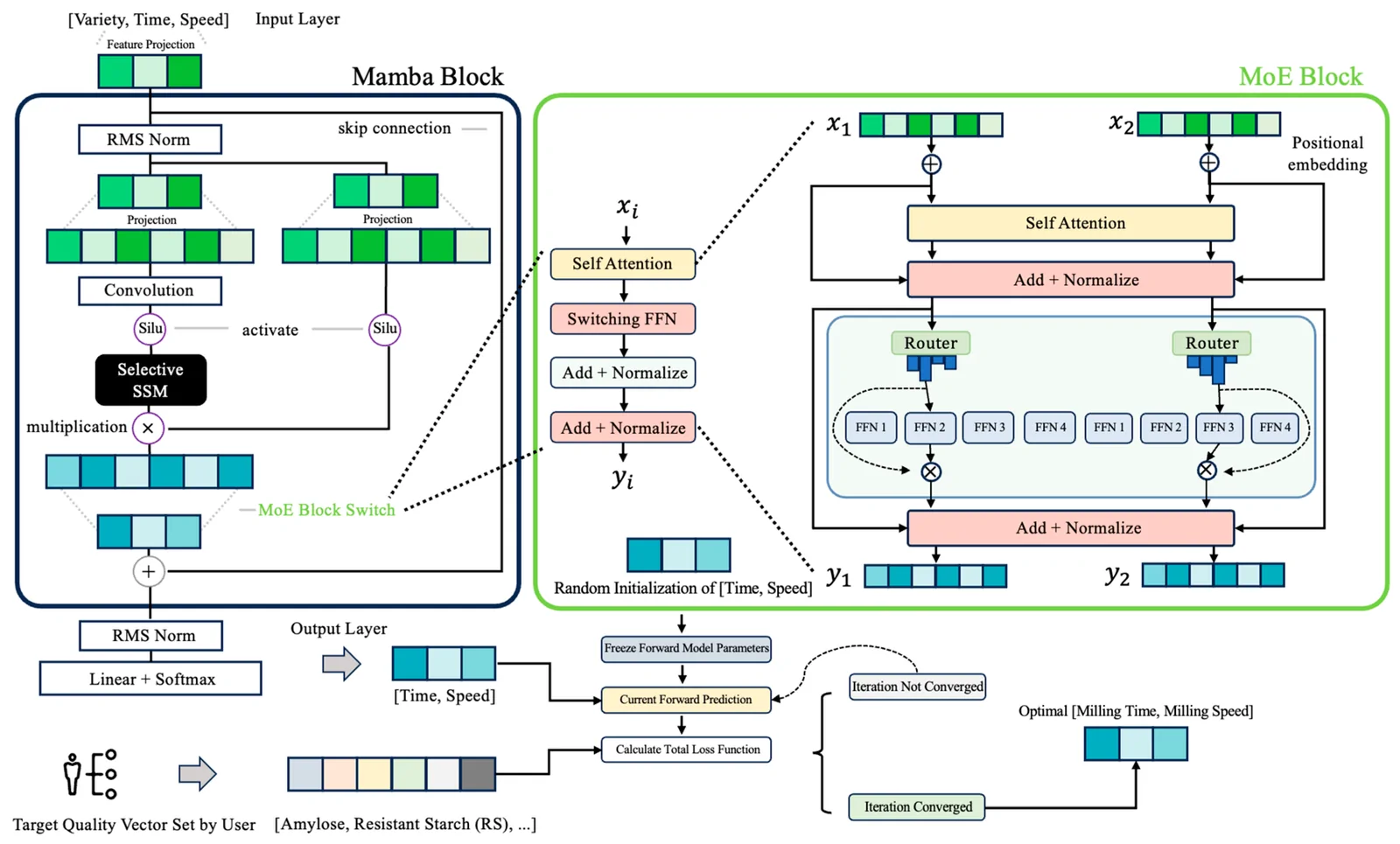
Schematic diagram of the RiceMambaOpt model overall architecture. Milling time, milling speed, and cultivar encoding are processed as fixed structured feature tokens; the Mamba hidden state represents learned feature interactions rather than an experimentally observed temporal or energy trajectory. Solid arrows show the forward data flow, dashed lines/arrows indicate routing and iterative feedback, and colors distinguish model modules and feature groups.

Rice milling is not an instantaneous mapping: the effects of milling duration and rotational speed accumulate as the outer layers of the kernel are progressively removed. The model inputs nevertheless consist of structured tabular variables, namely cultivar encoding, milling time, and milling speed, and do not constitute an intrinsically ordered temporal sequence. The Mamba architecture was therefore not assumed to possess an inherent temporal advantage over conventional tabular models; it was adopted as an empirical gated state-space feature extractor whose selective state-space operations provide nonlinear gated interactions among the process variables while remaining differentiable for gradient-based inverse optimization. As shown in Figure 4, cultivar encoding, normalized milling time, and normalized milling speed were represented as a fixed set of feature tokens, and recursive computation was performed using the discretized state equations in the Mamba module:

(2) $$h_{t}=\bar{A}h_{t-1}+\bar{B}x_{t}$$

This architecture enables the hidden state $h_{t}$ to retain and update the accumulated process-exposure state from the initial stage of processing to the current time point, thereby mathematically simulating the continuous cutting action of the rice mill on the grain surface. The hidden state is an abstract learned representation of cumulative processing exposure and should not be interpreted as a measurement of mechanical energy, torque, power, or temperature.

Considering the pronounced differences in endosperm structure and mechanical strength among rice cultivars, such as the extremely soft texture of glutinous rice and the harder, more brittle nature of *indica* rice, a single shared-parameter network is unlikely to accommodate multiple fracture patterns simultaneously. Therefore, a sparse Mixture of Experts (MoE) module was embedded after the Mamba layer. This module consists of multiple parallel expert networks, and a gating network dynamically computes routing weights according to the input cultivar encoding. For example, when the input corresponds to the high-hardness cultivar Luhui 6684, the gating network automatically activates the expert pathway specialized for high-shear-force responses while suppressing pathways designed for softer rice. This mechanism substantially improves predictive accuracy in multi-cultivar mixed scenarios. In the final output layer, a heteroscedastic regression strategy was adopted to simultaneously predict the expected values and variances of the quality attributes, thereby quantifying model uncertainty. The implemented RiceMambaOpt forward model used an 8-dimensional cultivar embedding and a 64-dimensional projection of the standardized continuous process variables. The projected features were processed by two consecutive Mamba blocks, each configured with a model dimension of 64, a state dimension of 16, an expansion factor of 2, a local convolution width of 4, and a dropout rate of 0.10. The resulting representation was passed to a sparse MoE layer containing three cultivar-adaptive expert networks. Each expert consisted of a 64–128–64 multilayer perceptron with SiLU activation, and top-1 routing with a capacity factor of 1.25 was used. The output head produced 18 values, comprising the predicted mean and log variance for each of the nine quality attributes. The training objective was the heteroscedastic Gaussian negative log-likelihood combined with a router load-balancing term weighted by 0.01. Model parameters were initialized using Xavier initialization and optimized using AdamW with an initial learning rate of 1 × 10^−3^, a weight decay of 1 × 10^−4^, and a batch size of 64. The learning rate followed a cosine decay schedule to 1 × 10^−5^ over a maximum of 500 training epochs, early stopping was applied after 40 consecutive epochs without improvement in real-validation loss, and the gradient norm was clipped at 1.0. Random seeds of 2024–2028 were used for data partitioning, parameter initialization, and model training, and the same hyperparameter configuration was used in all cross-validation folds. Complete architecture and training settings are reported in Appendix A—Table A4.

All architectural comparisons used identical real-observation partitions, target definitions, preprocessing procedures, training-only augmentation, and early-stopping criteria. Hyperparameters were selected using the corresponding training and validation partitions, while the independent real-sample test set remained excluded from model selection. The compared models and their selected configurations are reported in Appendix A—Table A5, and the results of ablation experiments are illustrated in Appendix A—Table A6.

#### 2.4.2. Inverse Solution of Process Parameters Under Physical Constraints

Based on the above framework, a high-accuracy forward prediction model was obtained:

(3) $$Y=F_{\theta }\left(X\right)$$

The resulting model was then treated as a differentiable evaluator for solving the optimal process parameters through inverse optimization. Specifically, all parameters θ of the forward model were first frozen, and the input milling process vector x was initialized as an optimizable random variable. Next, a total inverse optimization loss function, L, was defined, and X was iteratively updated using a gradient descent algorithm until the loss function converged. To prevent purely data-driven optimization from producing pseudo-solutions, which refer to mathematically fitting but practically infeasible parameter combinations such as extremely short milling times combined with very low milling speeds, a process-feasibility constraint term was incorporated into the loss function. For each target quality vector, 32 initial process vectors were independently sampled from uniform distributions within the equipment ranges of 0–60 s for milling time and 0–1050 r/min for milling speed. The process vectors were optimized using Adam with a learning rate of 0.05 for a maximum of 2000 iterations. After every update, the variables were projected back into the equipment-feasible bounds. The constraint coefficient *λ* was fixed at 10.0, and the minimum cumulative milling exposure was set to 160 nominal spindle revolutions. Optimization was terminated when the relative change in total loss remained below 10^−6^ for 50 consecutive iterations or when the maximum iteration number was reached. The feasible solution with the lowest total loss among the 32 initializations was retained. The total loss function was defined as follows:

(4) $$L\left(x\right)={\left|\left|F_{\theta }\left(x\right)-y^{*}\right|\right|}_{2}^{2}+\lambda \cdot L_{feas}\left(x\right)$$

where the first term measures the Euclidean distance between the predicted quality attributes under the current process conditions and the target quality vector $y^{*}$, whereas the second term, $L_{feas}\left(x\right)$, represents the normalized process-dose feasibility penalty. This formulation is consistent with hybrid machine-learning optimization strategies in which a trained predictive model is embedded within an iterative search procedure to guide feasible decision making [32]. The process-dose penalty is a heuristic regularization term based on the two controlled operating variables and should not be interpreted as a validated physical law. The milling-time and rotational-speed limits are separately enforced as hard equipment-domain constraints.

Given that the milling effect is fundamentally determined by the combined exposure to milling duration and rotational speed, a dimensionless normalized process-dose proxy was defined as follows:

(5) $$N_{rev}=\frac{x_{time}\cdot x_{speed}}{60}$$

where $x_{time}$ is measured in seconds and $x_{speed}$ is measured in revolutions per minute; therefore, $N_{rev}$ represents the nominal cumulative number of spindle revolutions. In the present study, $N_{rev}$ was used only as an empirical proxy for cumulative milling exposure under the same laboratory mill and standardized experimental setup. It should not be interpreted as mechanical energy. Calculation of actual mechanical work would require torque or instantaneous power measurements, for example, $W=\int \tau \left(t\right)\omega \left(t\right)\;dt$, which were not collected in the present study. Therefore, direct torque and power monitoring should be incorporated in future work to establish a physically dimensioned energy constraint.

When the parameter combination identified by the model produced a normalized process-dose value lower than the minimum threshold $D_{min}$ required for basic bran removal, the penalty term was activated:

(6) $$L_{feas}=\mathrm{ReLU}\left(N_{min}-N_{rev}\right)$$

where the value of $N_{min}$ was determined through preliminary calibration experiments conducted independently of model training and evaluation. Four cumulative milling-exposure levels, corresponding to 80, 120, 160, and 200 nominal spindle revolutions, were evaluated for each cultivar, with three independent milling runs performed at each exposure level. For every run, residual bran coverage was quantified by image segmentation using 100 randomly selected kernels, and basic bran removal was operationally defined as a mean residual bran coverage of no more than 10%. At exposure levels of 80, 120, 160, and 200 revolutions, the residual bran coverage values were 18.9 ± 1.7%, 10.8 ± 1.2%, 6.8 ± 0.9%, and 4.3 ± 0.7%, respectively, for Yangfunuo 4; 20.6 ± 1.9%, 11.9 ± 1.4%, 7.6 ± 1.1%, and 4.8 ± 0.8%, respectively, for Qiuguang; and 23.8 ± 2.1%, 13.6 ± 1.7%, 9.2 ± 1.3%, and 5.6 ± 0.9%, respectively, for Luhui 6684. Because 160 revolutions was the lowest tested exposure level at which all three cultivars satisfied the residual-bran-coverage criterion, a conservative common threshold of $N_{min}=160$ revolutions was adopted. This value corresponds to $x_{time}\times x_{speed}=9600\;s\cdot r/min$ under the former notation. The selected threshold is therefore an empirical process-feasibility threshold specific to the laboratory mill and experimental conditions used in this study, rather than a universal physical constant.

This term forces the optimization trajectory to move toward the empirically defined process-feasibility region. Through this process-dose- and equipment-constrained optimization mechanism, RiceMambaOpt is capable of identifying the optimal combination of milling time and milling speed that meets the desired nutritional and sensory requirements and is practical for real-world processing. The value of $N_{min}$ is an equipment-, material-, and operating-domain-specific empirical threshold rather than a universal physical constant. Similar $N_{rev}$ values indicate only model-implied similarity in cumulative time–speed exposure and do not establish equal torque, power, mechanical work, specific energy, frictional loss, temperature rise, or physical milling effect across cultivars.

#### 2.4.3. Implementation and Reproducibility Details

All computational experiments were implemented in Python 3.10.13 using PyTorch 2.2.1 and CUDA 12.1 and were executed on a single NVIDIA GeForce RTX 3090 GPU with 24 GB memory. The development–holdout partition used a random seed of 2024, and five complete training repetitions used seeds 2024, 2025, 2026, 2027, and 2028. NumPy, Python, and PyTorch random-number generators were initialized with the corresponding seed. Deterministic PyTorch algorithms and deterministic cuDNN execution were enabled. Hyperparameter selection and early stopping were based exclusively on performance for real validation samples. No holdout sample was used for preprocessing, generator fitting, hyperparameter selection, early stopping, variance calibration, or model selection. Since the output head predicts a mean and a log variance for each quality attribute, two-sided prediction intervals were constructed from the corresponding Gaussian quantiles, and their calibration was assessed on the untouched holdout set by comparing the nominal and empirical coverage probabilities at the 80%, 90%, and 95% levels together with the normalized mean interval width. The complete architecture, optimization settings, split indices, software environment, and random seeds are provided in Appendix A—Table A4 and the accompanying reproducibility repository.

### 2.5. Prospective Experimental Validation of Inverse Recommendations

The forward-prediction model and inverse optimizer were finalized before the prospective experiment was initiated. Twelve target quality profiles covering digestibility-oriented, sensory-oriented, and balanced objectives were specified before the corresponding processing conditions were generated. Four target profiles were established for each of the three rice cultivars, and RiceMambaOpt generated one feasible milling condition for each profile.

After the target profiles and recommended processing conditions had been fixed, new rice samples were prepared using reserved material from the same three cultivar lots. Each recommended condition was independently implemented three times using the laboratory-scale precision rice mill described in Section 2.1, resulting in 36 newly milled samples. These samples were not used for TabDDPM fitting, synthetic-sample generation, forward-model training, hyperparameter selection, inverse-optimizer development, or model recalibration. Starch digestibility and sensory attributes were measured using the same analytical and sensory-evaluation procedures described in Section 2.1.

Target attainment was evaluated using output-specific tolerances specified before examination of the experimental results. Agreement between the target and experimentally measured quality profiles was summarized using the proportion of profiles meeting all prespecified tolerances, RDS mean absolute error (MAE), mean sensory-score MAE, and joint normalized root-mean-square error (nRMSE). The complete target definitions, recommended milling conditions, experimental measurements, and profile-level errors are provided in Appendix A—Table A7.

## 3. Results and Discussion

### 3.1. Accuracy Evaluation and Comparative Analysis of the Forward Prediction Model

The reliability of inverse process optimization fundamentally depends on the fitting accuracy of the forward model, specifically on its ability to accurately establish the mapping from process parameters to multidimensional quality attributes in rice processing. To comprehensively evaluate the performance of the RiceMambaOpt architecture in capturing the nonlinear relationships between processing conditions and quality outcomes, this study rigorously selected nine core quality indicators as prediction targets, covering physicochemical composition, nutritional digestibility, and sensory eating quality. Specifically, these indicators included amylose content, which reflects cultivar-specific genetic characteristics; rapidly digestible starch (RDS), slowly digestible starch (SDS), and resistant starch (RS), which characterize the in vitro starch-digestibility profile and serve only as indirect laboratory proxies relevant to glycemic potential; and aroma, appearance, palatability, taste, and texture of cooled rice scores, which were recorded as trained-panel analytical sensory scores and do not by themselves establish consumer acceptance.

To validate the superiority of the proposed model, a broad set of baseline models was included for comparison, comprising the traditional machine learning algorithms SVR, RF, and XGBoost [33], together with quadratic response surface methodology (RSM), Gaussian process regression (GPR), and CatBoost, as well as the mainstream deep learning models MLP, CNN-LSTM, and the standard Transformer [34]. All models were trained and evaluated using the same leakage-controlled five-fold cross-validation partitions. Within each outer fold, hyperparameter selection was performed by three-fold inner cross-validation using only the 320 real training samples and synthetic samples generated by the corresponding training-fold TabDDPM. The 80 real samples in the outer validation fold and the 100-sample independent holdout set were not used for hyperparameter selection, and the mean coefficient of determination was used as the primary evaluation metric. This benchmark selection is consistent with recent tabular-learning studies showing that neural architectures and boosted-tree models should be compared under matched data regimes rather than treated as universally superior alternatives [35,36]. For each tunable model, 50 candidate configurations were evaluated within each outer training fold, and the configuration maximizing the mean R^2^ across the nine predicted quality attributes was selected. Single-output algorithms were fitted separately to each of the nine attributes using the same selected configuration. Neural-network training was stopped according to real inner-validation performance. The complete search spaces and selected configurations are reported in Appendix A—Table A8.

**Figure 5:**
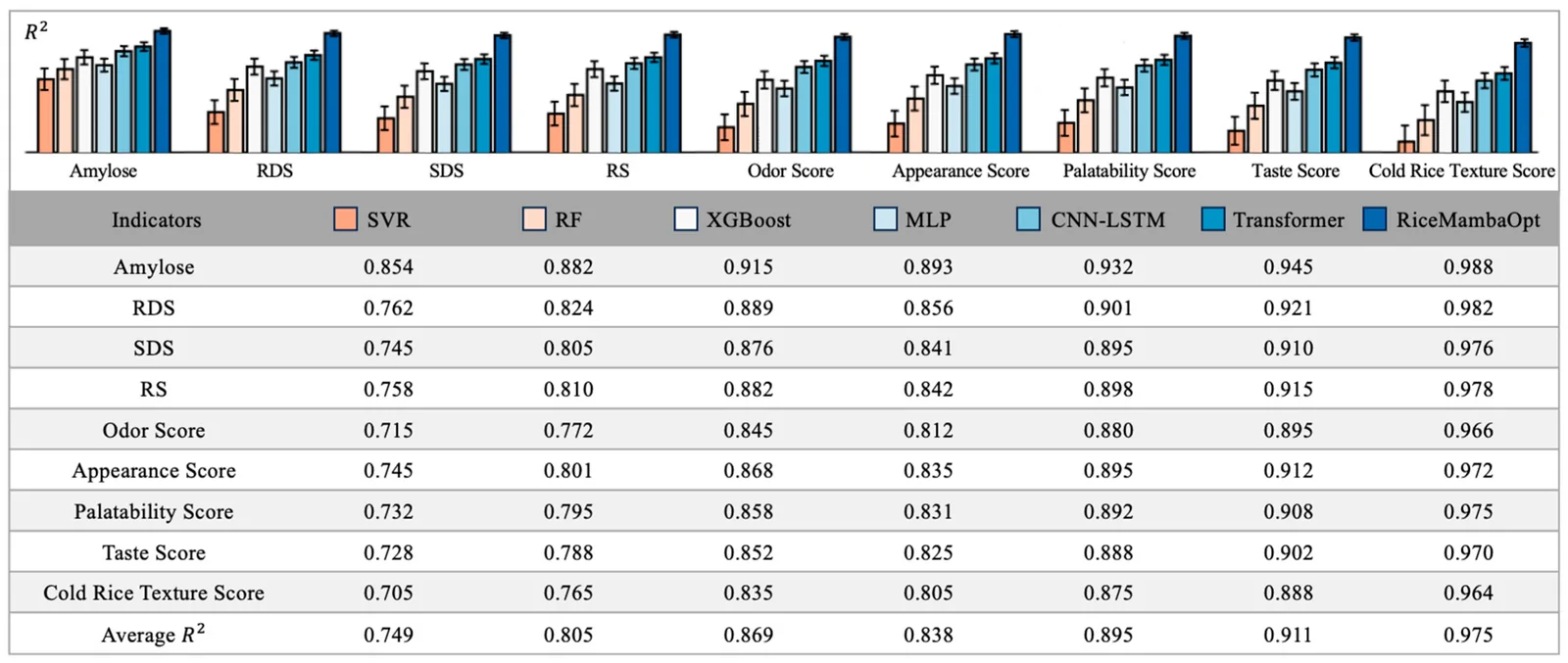
Comparison of prediction accuracy (*R*^2^) of different models for nine key quality indicators. All reported performance values were calculated using exclusively real experimental observations in the corresponding validation folds; synthetic observations were restricted to model training. Detailed fold-level and indicator-specific results are provided in Appendix A—Table A9 and Table A10.

**Figure 6:**
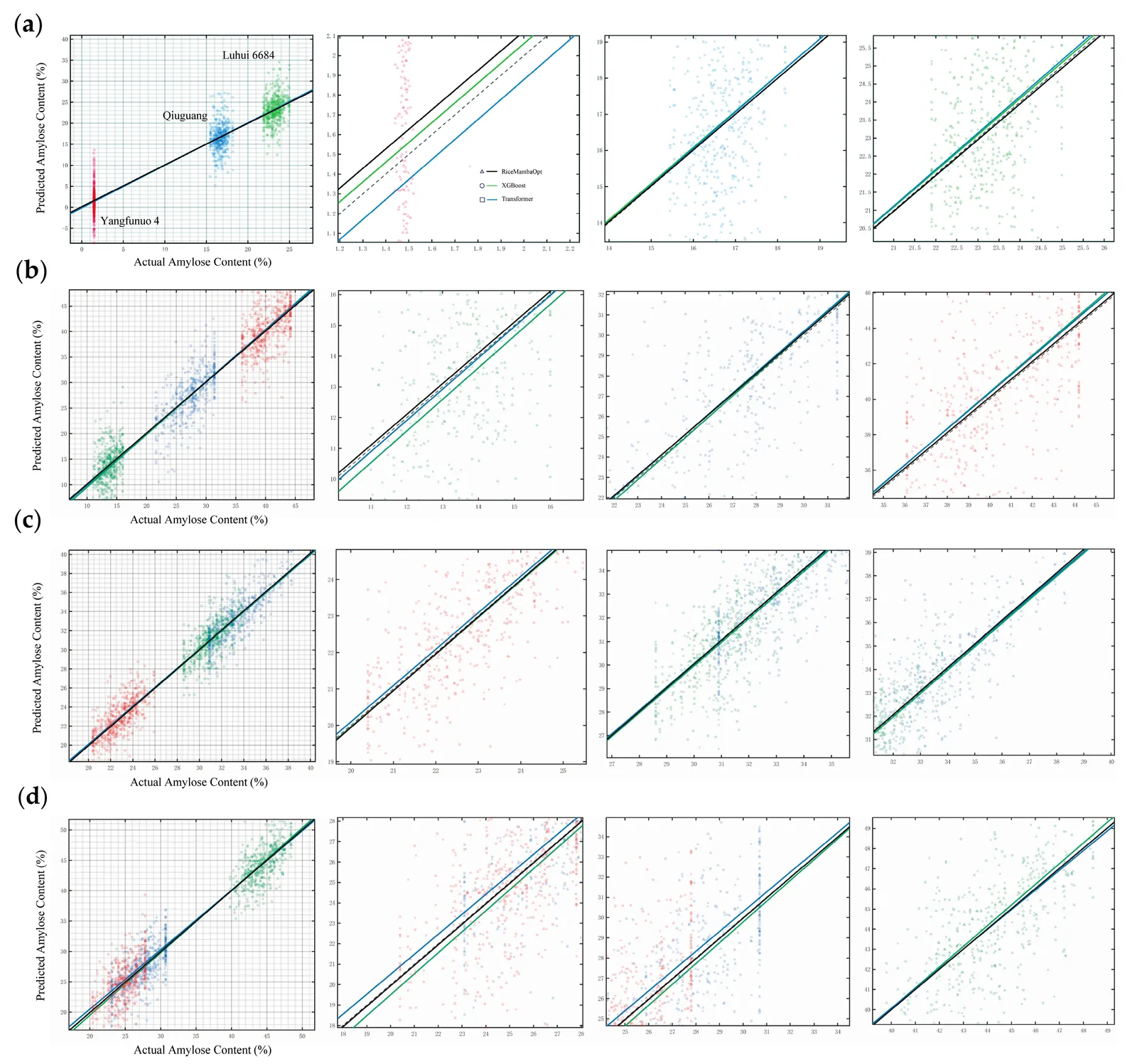
Density-based comparison of fitting performance among different models. (**a**) Scatter fitting prediction of amylose content; (**b**) scatter fitting prediction of RDS content; (**c**) scatter prediction results; (**d**) error distribution results. In each panel, the leftmost plot shows the predicted-versus-measured distribution for all samples, with the three cultivars (Yangfunuo 4, Qiuguang, and Luhui 6684) distinguished by color and overlapping observations drawn semi-transparently so that the local point concentration remains visible; the black line denotes the 1:1 identity line. The three magnified plots to the right resolve the cultivar-specific clusters and compare the fitted relationships obtained for RiceMambaOpt, XGBoost, and the Transformer baseline. All reported values were calculated using exclusively real experimental observations in the corresponding validation folds; synthetic observations were restricted to model training. Dashed lines show the model-specific fitted relationships; the black solid line is the 1:1 identity line.

As shown in Figure 5, traditional machine learning models such as SVR and RF performed reasonably well when predicting physicochemical indicators with relatively strong linearity, such as amylose content. However, their performance reached a clear bottleneck when predicting attributes such as hardness and viscosity, which are strongly influenced by the dynamic mechanical effects of the milling process ($R^{2}$ < 0.8). Owing to its powerful ensemble-learning capability, XGBoost achieved higher accuracy than MLP, but it still struggled to capture the long-range cumulative energy characteristics of the process. In contrast, CNN-LSTM and Transformer models, which incorporate temporal modeling capability, showed markedly improved performance, with the average $R^{2}$ exceeding 0.90. Nevertheless, the RiceMambaOpt model achieved the best performance across all nine indicators. In the expanded matched comparison, the mean $R^{2}$ values were 0.781 ± 0.045 for quadratic RSM, 0.749 ± 0.043 for SVR, 0.805 ± 0.034 for RF, 0.838 ± 0.026 for MLP, 0.858 ± 0.030 for GPR, 0.869 ± 0.025 for XGBoost, 0.895 ± 0.016 for CNN-LSTM, 0.904 ± 0.020 for CatBoost, 0.911 ± 0.016 for Transformer, and 0.975 ± 0.008 for RiceMambaOpt. RiceMambaOpt achieved a higher indicator-specific mean $R^{2}$ than Transformer and CatBoost for all nine outputs. Two-sided exact Wilcoxon signed-rank tests yielded p=0.0039 for both comparisons, and both remained significant after Holm correction (adjusted p=0.0078). In particular, for subjective sensory attributes such as palatability score, its $R^{2}$ remained above 0.96, demonstrating an exceptional ability to characterize both the physical milling process and the associated sensory responses. To more intuitively illustrate the predictive stability and error distribution of each model, a multidimensional comparative visualization was constructed (see Figure 6).

On the untouched real holdout set, the per-variable *R*^2^ values of RiceMambaOpt ranged from 0.963 for aroma to 0.987 for amylose content, with a mean value of 0.975. The corresponding nRMSE values ranged from 4.08% to 5.50%, with a mean of 4.52%. The lowest nRMSE was obtained for amylose content, whereas the largest value was obtained for taste score. Importantly, RiceMambaOpt achieved the lowest nRMSE for every output variable when compared with all traditional machine-learning and deep-learning baselines. Complete per-variable results are provided in Appendix A—Table A11 and Table A12. The heteroscedastic prediction intervals were also well calibrated on the same holdout set: the empirical coverage probabilities were 79.0%, 88.4%, and 93.1% at the nominal 80%, 90%, and 95% levels, corresponding to a mean absolute coverage error of 1.5 percentage points, and the complete calibration metrics are reported in Appendix A—Table A13. Under identical data partitions, target definitions, and equipment bounds, the matched quadratic RSM reached a mean forward-prediction nRMSE of 16.26% against 4.52% for RiceMambaOpt, and its holdout inversion errors were correspondingly larger (milling time, 5.840 s versus 0.960 s; milling speed, 86.700 r/min versus 19.522 r/min); the complete item-by-item comparison is given in Appendix A—Table A14.

The sensitivity analysis further showed that model performance did not arise solely from the architecture. When the forward model was trained using only the real training samples, without TabDDPM augmentation, its mean *R*^2^ decreased to 0.932 ± 0.015 and its mean nRMSE increased to 8.74%. With the selected 9:1 synthetic-to-real training ratio, mean *R*^2^ increased to 0.975 ± 0.008 and mean nRMSE decreased to 4.52%. The improvement was observed for all nine output variables, although its magnitude varied among physicochemical and sensory attributes. These results indicate that leakage-controlled synthetic augmentation contributed to predictive performance within the observed data domain. They do not imply that synthetic observations can replace external experimental validation. Per-variable results are reported in Appendix A—Table A15.

### 3.2. Validation of Process Inversion Accuracy Using an Independent Test Set

To objectively evaluate the practical within-domain accuracy of the RiceMambaOpt model, a strict holdout validation strategy was adopted in this study. Before model training, 100 samples were randomly selected from the original dataset to construct an independent test set. This split was performed before fitting TabDDPM, generating any synthetic samples, defining cross-validation folds, or optimizing the hyperparameters of the forward model. The remaining 400 real observations constituted the development set used for leakage-controlled five-fold cross-validation and final model training. This dataset was never involved in either TabDDPM-based data augmentation or parameter updating of the MoE-Mamba model, thereby ensuring an independent assessment of model performance for samples drawn from the same experimental domain. However, as the holdout samples originated from the same three cultivars, laboratory equipment, geographical source, harvest context, and limited collection of raw-material batches as the development samples, this evaluation should not be interpreted as external validation across seasons, regions, batches, or industrial milling systems. First, the true quality attribute vectors of the test samples ($Y_{true}$) were used as target inputs to the trained RiceMambaOpt system. Next, under the guidance of process-feasibility constraints, the model inversely inferred the recommended process parameters (${\hat{X}}_{pred}$). Finally, the recommended parameters were compared one by one with the actual process parameters corresponding to each sample ($X_{true}$), and the mean absolute error (MAE) and coefficient of determination ($R^{2}$) were calculated.

**Figure 7:**
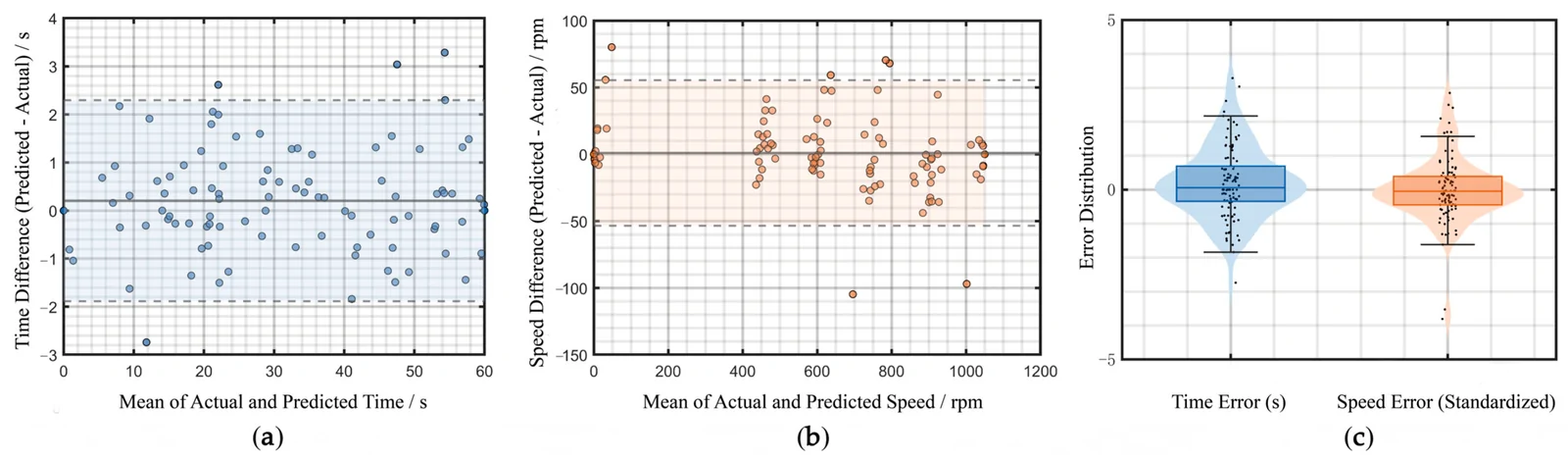
Agreement and error distribution analysis of inverse prediction results for milling time and milling speed. (**a**) Bland–Altman agreement plot for milling time; (**b**) Bland–Altman agreement plot for milling speed; (**c**) violin and box plots showing the distributions of milling-time error and standardized milling-speed error. Blue and orange identify milling-time and milling-speed errors, respectively; dots are sample-wise differences, solid horizontal lines are mean biases, dashed lines are the 95% limits of agreement, and shaded bands span those limits. In panel (**c**), violin areas show error density, boxes show the interquartile range and median, and black dots are individual samples.

The results demonstrated that the model exhibited strong recovery capability and stable within-domain performance in inverse process prediction. Statistical analysis showed (see Figure 7) that, on the independent test set, the mean absolute error between the inversely predicted milling time and the true experimental setting was only 0.960 s, with an $R^{2}$ of 0.986. For milling speed, the mean absolute error was 19.522 r/min, with an $R^{2}$ of 0.851. The prediction accuracy for milling time was markedly higher than that for milling speed, which may be attributed to the fact that, during rice milling, the contribution of time to bran removal is strongly linear and dominant, whereas the effect of speed on quality exhibits a nonlinear plateau effect. In other words, within a certain high-speed range, fluctuations in milling speed have only a limited influence on the final quality, resulting in a reasonable tolerance in speed inversion. Overall, both the time and speed errors fell within the control precision range of industrial rice milling equipment, indicating that the inversion errors were compatible with the nominal adjustment resolution of milling equipment under the studied laboratory conditions. This comparison does not by itself demonstrate equivalent performance on other cultivars, harvest years, geographical origins, raw-material batches, or industrial mills.

Notably, although the predicted milling speeds of some samples deviated numerically from the true values, secondary verification using the forward model showed no significant difference between the corresponding predicted quality attributes and the target values. This result suggests the presence of a model-derived time–speed dose equivalence within the observed experimental domain: different combinations of milling time and speed may produce similar predicted quality profiles when their normalized time–speed doses and resulting degrees of bran removal are comparable. A higher-speed, shorter-duration condition may provide a greater instantaneous shear and collision rate, whereas a lower-speed, longer-duration condition may provide a lower instantaneous intensity over a longer grain–mill contact period. These distinct trajectories can produce similar final quality attributes even though their transient mechanical and thermal histories may differ. However, this observation should not be interpreted as proof of true mechanical-energy equivalence because torque, instantaneous power, friction coefficient, sample temperature, and heat loss were not measured. Direct comparison of energy efficiency among alternative parameter combinations will require simultaneous torque, power-consumption, and temperature measurements.

### 3.3. Analysis of Differences in Process Optimization Potential Among Cultivars

Using the validated RiceMambaOpt system, this study defined a highly challenging composite target vector, representing the simultaneous pursuit of superior eating quality and a lower in vitro RDS proportion, with the constraints of a palatability score exceeding 20 and an RDS value below 20%, in order to evaluate the processing adaptability of rice cultivars with different genetic backgrounds. The inverse optimization results for the three cultivars are presented in Table 2, clearly revealing the interdependent relationships among cultivar, process, and quality. Qiuguang, the *japonica* cultivar, exhibited the best processing adaptability and was the only cultivar for which feasible optimization was achieved. For this cultivar, the model did not simply push the process to the operational limits of the equipment; instead, it intelligently identified a process combination of high milling speed and moderate milling time. Specifically, the model recommended a high speed of 1020 r/min to generate sufficient shear force for the rapid removal of coarse fibrous components from the outer layer of brown rice, thereby substantially improving palatability to a high level of 26.5. At the same time, the milling time was strictly controlled within a moderate range of 38.5 s. This strategy effectively prevented the complete exposure and excessive gelatinization of deeper starch granules caused by overprocessing [37], thereby successfully maintaining RDS at 21.6%, near the boundary of the desired range. Recent milling-degree studies likewise indicate that starch structure, digestibility, and cooking quality shift nonlinearly with bran removal intensity [38]. This precise balancing strategy suggests that, for *japonica* rice, fine-tuned physical process control has the potential to overcome the conventional trade-off between eating quality and the in vitro starch-digestibility profile. For context, representative response-surface studies of rice milling and rice drying have reported optimal conditions and responses obtained with different cultivars, equipment, and target variables; these are summarized alongside the present solution in Appendix A—Table A14, where the numerical outcomes are listed for reference only and are not directly comparable with those of the present study.

In contrast, the optimization results for Yangfunuo 4 revealed the limitations of physical processing in the face of inherent biological and genetic characteristics. Although the model explored multiple parameter combinations, it ultimately determined that the target was infeasible and instead provided a loss-minimizing strategy involving short-duration, light milling. This did not indicate model failure; rather, the extremely high amylopectin content of glutinous rice gives it a naturally high RDS baseline exceeding 35%, and with prolonged milling, the mechanical thermal effect further disrupts starch crystallites [39], causing RDS to increase rather than decrease. Therefore, the model selected a restrained optimization path, using only 18.0 s of milling to rapidly improve surface texture and stopping energy input once palatability just reached the acceptable threshold of 22.5. This result confirms at the data level that, for glutinous rice with a genetically predisposed high in vitro RDS profile, physical milling alone cannot achieve the prespecified low-RDS target under the current in vitro assay conditions and must be combined with other interventions, such as enzymatic modification. Recent evidence that germination combined with moderate milling can alter both textural quality and starch digestion characteristics in brown rice further supports the need for integrated processing strategies beyond milling alone [30].

The case of Luhui 6684 further highlights the model’s ability to warn against texture deterioration. Although this *indica* cultivar had a naturally low RDS of only 13.5%, fully meeting the prespecified laboratory RDS criterion, the model did not recommend an aggressive bran-removal strategy. Instead, it selected a gentle low-speed processing mode of 750 r/min. The reason is that the cultivar is characterized by high vitreousness and a hard kernel texture; under intense mechanical friction at high speed, the grain surface can rapidly become overly compacted, resulting in a sharp increase in hardness and a severe decline in eating quality [40]. Although the final predicted palatability score was 18.5, slightly below the target threshold, this value represented the highest achievable eating quality under the condition of preventing complete texture collapse. Therefore, RiceMambaOpt is not merely a parameter calculator; it is also capable of identifying the optimal trade-off between physical feasibility and biological limitation. The RDS value reported here describes only the response obtained using the selected in vitro digestion assay and should not be interpreted as evidence that the resulting rice has a clinically low GI.

### 3.4. Analysis of the Multi-Objective Trade-Off Between In Vitro Starch Digestibility and Sensory Quality

In practical applications within the rice processing industry, the pursuit of health-related attributes, such as a low glycemic index, is often inherently constrained by the desire for superior sensory palatability. To elucidate the coupling mechanism between these two objectives and identify the optimal processing window, this study selected Qiuguang as the target cultivar. As the *japonica* cultivar with the broadest processing adaptability, Qiuguang was further analyzed using the trained RiceMambaOpt model through high-density inverse simulations across the entire process space. This design follows the broader multi-objective data-driven paradigm in food processing, in which predictive models are coupled with optimization to balance conflicting quality, nutritional, and engineering objectives [41].

**Figure 8:**
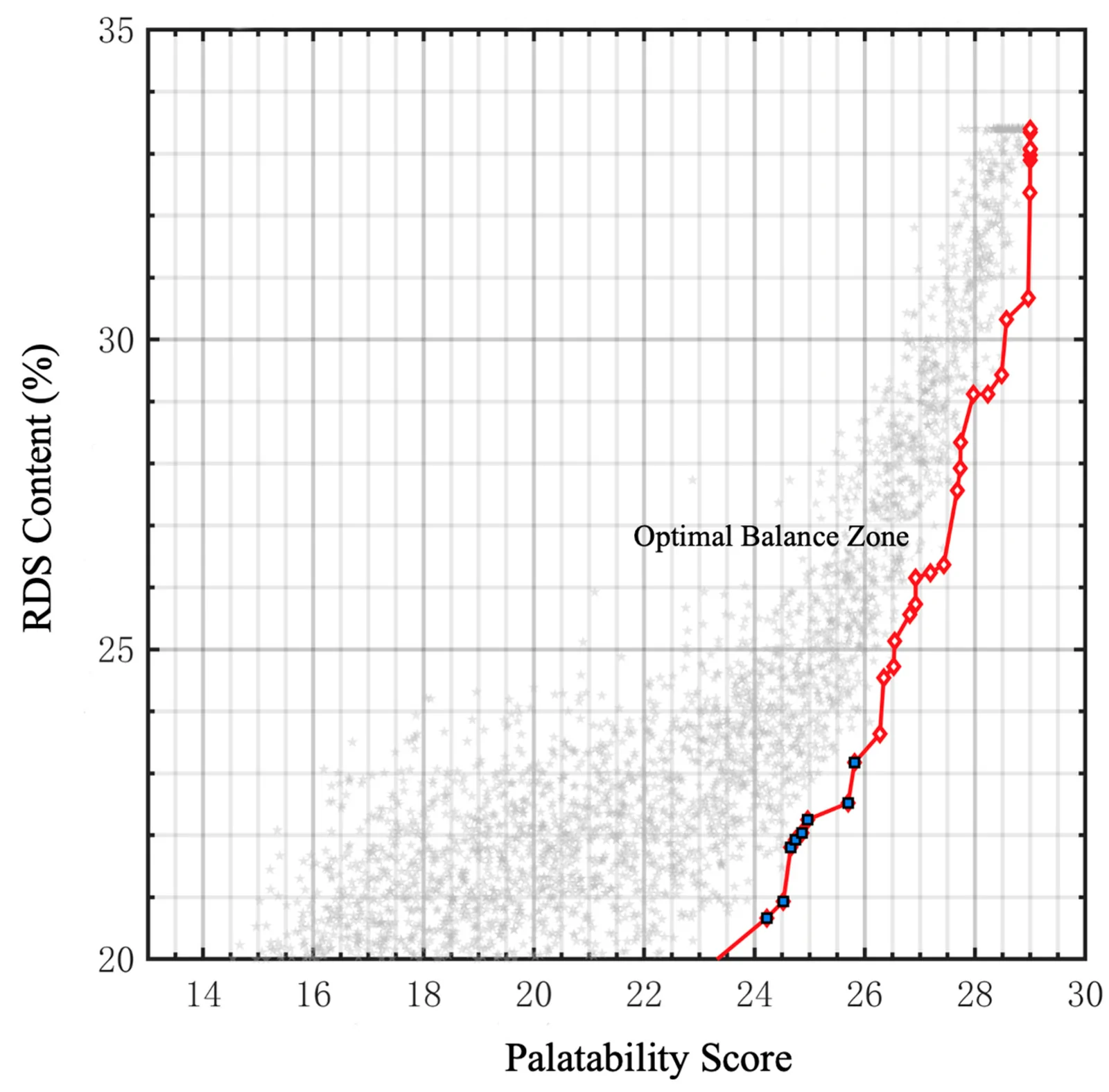
Pareto multi-objective trade-off frontier curve of RDS content and palatability score for the Qiuguang cultivar. Specifically, gray stars denote feasible predictions, open red diamonds mark Pareto-optimal solutions, and filled blue squares identify solutions in the selected balance zone.

By fixing milling speed at different gradient levels while continuously varying milling time, thousands of process-specific quality prediction data points were generated. These data were then used to construct a two-dimensional distribution map of rapidly digestible starch (RDS) content and palatability score (see Figure 8). The results revealed a typical nonlinear trade-off between the two variables: as palatability increased, RDS first rose gradually and then increased sharply in an exponential-like manner.

More specifically, when the palatability score was in the range of 15 to 24, the increase in RDS was relatively moderate and was mainly concentrated between 22% and 26%. This stage corresponded to the initial removal of the bran layer from brown rice, during which the reduction in dietary fiber substantially improved the rough mouthfeel, while the starch granules had not yet undergone severe internal mechanical damage. As a result, the negative impact on the laboratory-measured starch-digestibility profile remained limited, making this phase a high-return processing region. However, when the pursuit of superior eating quality pushed the palatability score beyond 26, the RDS content exceeded 30% and increased explosively. This indicates that achieving the final 10% improvement in sensory quality requires the application of extremely high mechanical energy, leading to severe physical disruption and thermal gelatinization of the starch crystalline structure and, consequently, a substantial increase in RDS under the current in vitro assay conditions.

Based on this Pareto frontier, this study precisely identified the processing “sweet spot” for the Qiuguang cultivar, namely the region in which the palatability score remained around 25 while RDS was controlled below 28%. This finding not only confirms the scientific necessity of moderate processing but also provides a quantitative basis for establishing differentiated product standards. Specifically, this interval may be considered a laboratory-defined processing region that balances a relatively lower in vitro RDS proportion with acceptable palatability. However, it should not be used to label the resulting product as low-GI or to infer a reduced metabolic risk until the prediction is confirmed using standardized in vivo glycemic-response testing.

### 3.5. Ablation Study of the Process-Feasibility Constraint Mechanism on the Feasibility of Process Inversion

To verify the decisive role of the process-feasibility constraint module in the RiceMambaOpt system during process inversion, a rigorous ablation study was conducted. An unconstrained inversion model was used as the control, in which the penalty terms for the minimum cumulative milling-exposure boundary and equipment limits were removed during inverse gradient search, and minimization of the quality prediction error was used as the sole objective. The *indica* cultivar Luhui 6684, which presented the greatest processing difficulty, was selected as the test material, and three highly representative extreme or composite quality targets were defined to evaluate model feasibility under complex scenarios.

To quantitatively evaluate the deviation between the optimized quality outputs and the predefined objectives, a normalized target-deviation error was calculated as:

(7) $$E_{NTD}=100\sqrt{m^{-1}\sum_{j=1}^{m}{\left[\left({\hat{y}}_{j}-y_{j}^{*}\right)/\left(y_{j,max}-y_{j,min}\right)\right]}^{2}}$$

where m=2 is the number of quality objectives in each scenario, ${\hat{y}}_{j}$ is the quality value predicted by the forward model for the inversely obtained process parameters, $y_{j}^{*}$ is the specified target-boundary value, and $y_{j,max}-y_{j,min}$ is the observed range of the corresponding quality indicator in the original real dataset. For inequality objectives, the specified threshold was treated as the target-boundary value. A lower $E_{NTD}$ value therefore indicates that the predicted quality vector is closer to the specified optimization objective.

The results are presented in Table 3. In the absence of process-feasibility constraints, the purely data-driven model tended to exploit the overfitting behavior of the neural network to identify numerical shortcuts. For example, in Scenario A, to preserve an extremely high level of resistant starch, the unconstrained model produced a completely infeasible milling speed of 7500 r/min combined with an extremely short milling time of 0.5 s, attempting to manipulate the loss function through an unrealistic time–speed combination; such a solution is entirely inoperable on physical equipment. In Scenario B, the model even generated a negative time parameter, indicating a severe logical hallucination.

**Figure 9:**
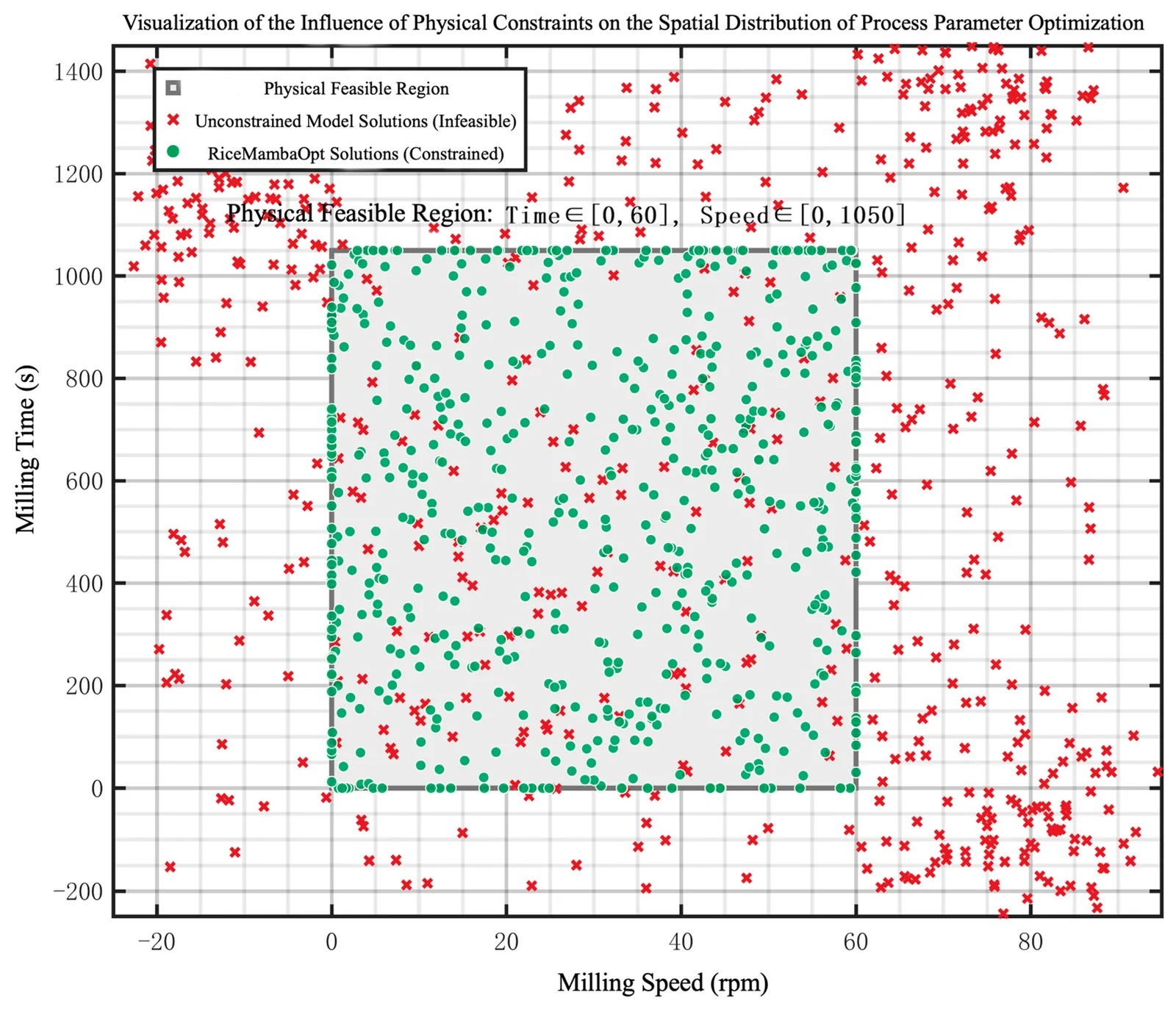
Visualization of the influence of physical constraints on the spatial distribution of process parameter optimization. Green dots denote constrained RiceMambaOpt solutions, red crosses denote unconstrained solutions, and the gray rectangle marks the feasible domain.

In contrast, after the introduction of the normalized process-dose penalty and equipment-domain constraints, the RiceMambaOpt system exhibited strong engineering rationality. When faced with the same challenging targets, the model no longer pursued mathematically perfect zero error; instead, it searched for the optimal solution within the effective operating range of the equipment (0–60 s, 0–1050 r/min). The corresponding normalized target-deviation errors were 3.4%, 7.6%, and 4.2% for Scenarios A, B, and C, respectively. These modest increases in objective-space error reflect the trade-off required to maintain engineering feasibility rather than a reduction in model accuracy. To further visualize this mechanism, a comparative map of the optimization trajectories of the two models in parameter space was constructed (see Figure 9).

As shown in Figure 9, the gray rectangular region represents the physically feasible domain of the actual rice mill (Time ∈ [0, 60], Speed ∈ [0, 1050]). The solutions produced by the unconstrained model (red crosses) were widely scattered outside of the feasible domain, showing obvious dispersion and a lack of controllability, whereas the solutions generated by RiceMambaOpt (green circles) were confined by the physical penalty term to within the rectangle or along its boundaries. This contrast provides strong evidence that RiceMambaOpt effectively integrates domain-specific physical knowledge into the deep learning framework, avoids the logical hallucination risks commonly associated with black-box models, and ensures that every recommended process condition has genuine practical feasibility.

### 3.6. SHAP-Based Interpretability Analysis of Key Process Factors

Finally, to gain deeper insight into the differential decision-making logic of the RiceMambaOpt system when handling rice cultivars with different genetic backgrounds, and to determine which quality requirements primarily governed the selection of process parameters for each cultivar, a hierarchical reverse SHAP analysis was conducted. Specifically, surrogate diagnostic models were separately constructed for Luhui 6684 (LH), Yangfunuo 4 (WX), and Qiuguang (QG) to map the nine quality indicators to milling speed and milling time.

For each cultivar, 10,000 physically feasible quality vectors were sampled within the empirical quality domain and submitted to the complete RiceMambaOpt inverse optimization pipeline. The corresponding recommended milling-time and milling-speed values were treated as reference outputs. Two gradient-boosted-tree surrogate regressors were then fitted for each cultivar, with 8000 samples used for surrogate training and 2000 independent samples reserved for fidelity evaluation. Surrogate fidelity was evaluated by calculating $R^{2}$, RMSE, and MAE between the surrogate-predicted process parameters and the corresponding process parameters returned by RiceMambaOpt. The fidelity $R^{2}$ values ranged from 0.951 to 0.975, with an overall mean of 0.966 across the six surrogate models. The complete fidelity results are provided in Appendix A—Table A16. These fidelity metrics measure the ability of the surrogate models to reproduce RiceMambaOpt’s inverse recommendations and should not be interpreted as experimental prediction-accuracy metrics.

**Figure 10:**
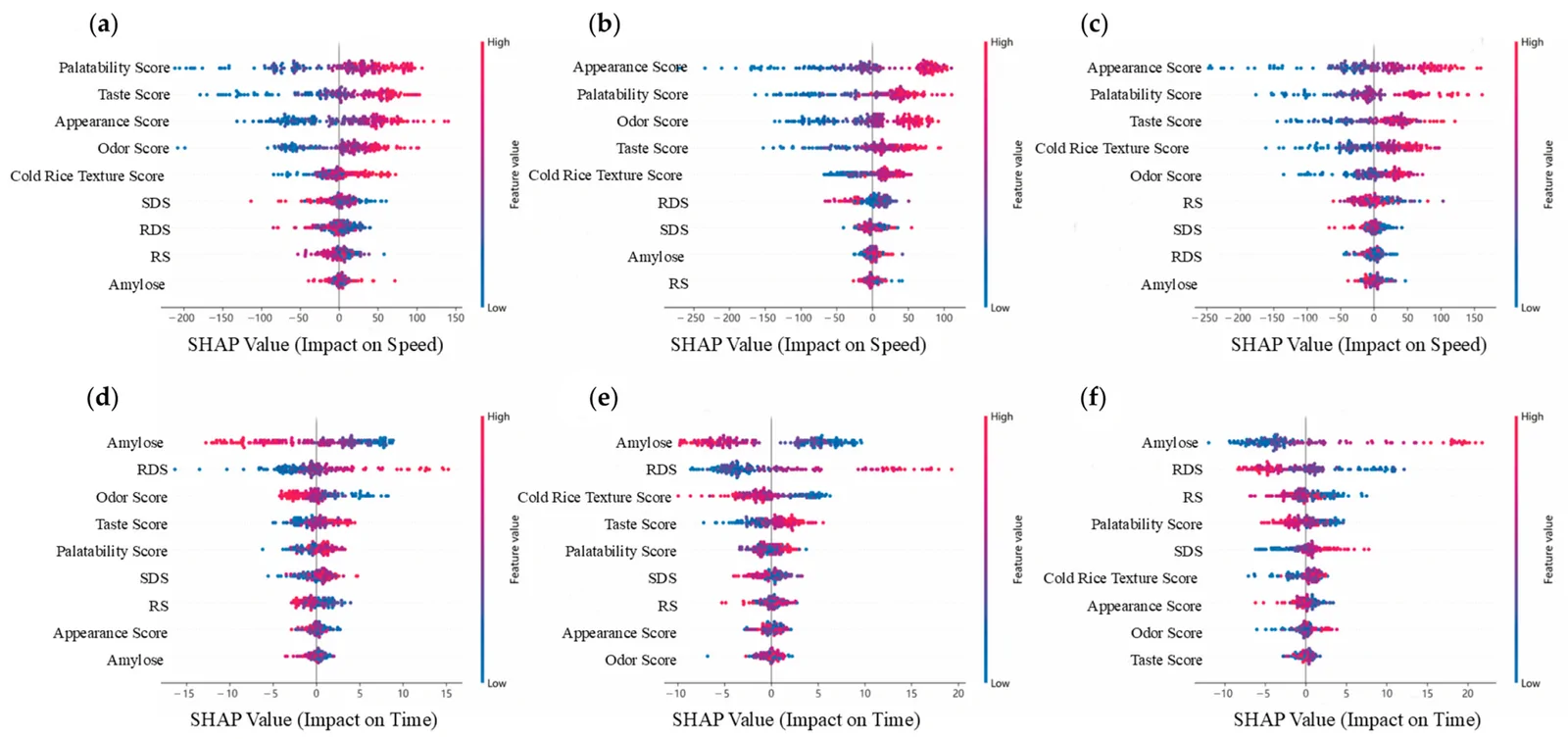
Surrogate-model-based reverse SHAP analysis spectrum of decision-driving forces for rice milling speed and time among different varieties. (**a**) Luhui 6684 (LH): SHAP summary plot for milling speed; (**b**) Yangfunuo 4 (WX): SHAP summary plot for milling speed; (**c**) Qiuguang (QG): SHAP summary plot for milling speed; (**d**) Luhui 6684 (LH): SHAP summary plot for milling time; (**e**) Yangfunuo 4 (WX): SHAP summary plot for milling time; (**f**) Qiuguang (QG): SHAP summary plot for milling time. SHAP values were calculated using six cultivar- and output-specific surrogate models with fidelity $R^{2}$ values ranging from 0.951 to 0.975 relative to the complete RiceMambaOpt inverse optimization pipeline.

SHAP values were then calculated to quantify the marginal contribution of each indicator to process-related decisions, consistent with the growing use of explainable AI techniques for interpreting food-engineering models [42]. Figure 10 presents a set of six SHAP summary beeswarm plots, which visually illustrate the differences in decision-driving factors across scenarios. The SHAP values quantify associations learned by the surrogate models and should not be interpreted as direct evidence of causal physicochemical mechanisms. The following mechanistic interpretation is therefore based on the observed SHAP patterns together with established knowledge of rice-bran abrasion, grain fracture, and starch responses to milling intensity.

Analysis of the milling speed decision maps in the first row of Figure 10a–c revealed that sensory quality played a dominant role in determining mechanical shear intensity, although the specific priorities differed markedly across cultivars because of genetic background. For the high-hardness *indica* cultivar Luhui 6684 (see Figure 10a), palatability score ranked first in feature importance, and a high demand for palatability (red sample points) markedly shifted the SHAP values in the positive direction. This indicates that, for this type of hard rice with high vitreousness, the model considers a sufficiently high milling speed necessary to generate strong shear force in order to effectively improve the rough surface texture and enhance eating quality. In contrast, for the glutinous cultivar Yangfunuo 4 (see Figure 10b) and the *japonica* cultivar Qiuguang (see Figure 10c), appearance score replaced palatability as the primary driver of increased milling speed. This suggests that, for cultivars with softer texture or a more rounded grain shape, increasing milling speed mainly contributes to bran removal and thereby improves whiteness and gloss rather than directly altering textural perception. Mechanistically, increasing rotational speed raises the frequency and relative velocity of grain–roller and grain–grain contacts. This increases the instantaneous abrasion and shear rate at the kernel surface, accelerates removal of the bran layer, and reduces surface roughness. These changes can increase whiteness and gloss and reduce the coarse mouthfeel associated with residual bran. The response is cultivar-dependent because a harder and more vitreous kernel can tolerate or require greater shear before comparable surface modification occurs, whereas softer glutinous or *japonica* kernels may exhibit visible surface changes at lower mechanical intensity. Excessive speed may nevertheless increase fissuring or surface compaction; therefore, the positive sensory association should not be interpreted as a monotonic benefit outside of the observed speed range.

Further analysis of the milling time decision maps in the second row of Figure 10d–f showed that the logic governing process duration was highly consistent across cultivars and was primarily controlled by two physicochemical indicators, namely amylose content and rapidly digestible starch (RDS). In all three cultivars, the SHAP distributions of RDS exhibited a clear positive trend: red sample points representing high RDS values were concentrated on the right side of the *x*-axis (positive SHAP values), whereas blue sample points representing low RDS values were concentrated on the left side (negative SHAP values). This pattern clearly reflects the mechanism of time- and heat-related effects captured by the model. Specifically, to satisfy the model objective of reducing RDS under the current in vitro digestion assay, the model tends to shorten milling time to prevent excessive thermal accumulation caused by prolonged mechanical friction, which would otherwise lead to excessive gelatinization of starch crystallites. Unlike rotational speed, milling time directly extends the duration of grain contact with the abrasive surfaces. A longer treatment can therefore increase cumulative bran removal, expose deeper endosperm regions, and prolong repeated compression, friction, and impact on starch-containing tissues. These effects may increase mechanical damage to starch granules and reduce structural barriers to enzymatic hydrolysis, thereby increasing the measured RDS fraction. Prolonged friction may also increase sample temperature and promote additional disruption of ordered starch regions. However, sample temperature and starch crystallinity were not monitored dynamically in this study; consequently, thermal accumulation and crystalline disruption are proposed mechanistic explanations rather than directly measured causal intermediates.

Taken together, comparison of the upper and lower rows indicates that RiceMambaOpt developed a clearly differentiated decision-making framework during process inversion. Milling speed was primarily used to regulate sensory experience, with palatability and appearance as the core targets, and its adjustment strategy varied flexibly according to cultivar hardness. By contrast, milling time was more strictly anchored to the in vitro starch-digestibility attributes centered on RDS. This decoupled decision-making capability, grounded in both observed physicochemical and sensory associations, indicates that the model goes beyond numerical fitting and is able to accurately characterize the complex relationships between process conditions and quality formation during rice processing. A plausible interpretation is therefore that speed primarily controls the instantaneous mechanical pathway governing surface abrasion and sensory modification, whereas time controls the cumulative exposure pathway governing bran-removal depth, starch damage, and the measured in vitro digestibility profile. The two variables are not completely independent: both contribute to the normalized time–speed dose, and different combinations may converge to similar endpoint qualities through different instantaneous shear and thermal histories. Verification of this interpretation will require future experiments that simultaneously record torque, electrical power, kernel or flour temperature, bran-removal rate, starch crystallinity, and granule damage during milling.

## 4. Conclusions

In this study, the RiceMambaOpt process optimization system was developed, and a conditional tabular diffusion model (TabDDPM) was introduced to address the problem of sparse data distribution under small-sample conditions. The results showed that the system achieved a mean coefficient of determination ($R^{2}$) of 0.975 across nine core indicators spanning physicochemical, nutritional, and sensory attributes, significantly outperforming baseline models such as the Transformer. The model accurately quantified the nonlinear effects of milling time, rotational speed, and their normalized process-dose proxy on multidimensional quality attributes, thereby establishing an algorithmic benchmark for digital twin modeling and high-precision simulation of rice processing. At the mechanistic level, this study used reverse SHAP analysis and Pareto-based multiobjective analysis to successfully disentangle the differential process decision-making mechanisms of rice cultivars with distinct genetic backgrounds. The results revealed that appearance score and RDS constitute a pair of key competing levers driving process decisions and quantitatively identified the optimal balance window between palatability and health-related attributes for the Qiuguang cultivar. Further hierarchical analysis demonstrated that the model had learned the cultivar-specific response patterns represented in the observed data. This direction is aligned with recent digital-twin research in food systems, which emphasizes the integration of predictive models, process simulation, and decision support for real-time optimization [43,44]. Since torque, power, sample-mass-normalized energy, frictional losses, and temperature were not measured, the present process-dose proxy should not be interpreted as mechanical energy or used to support conclusions regarding energy efficiency or thermal mechanisms.

## Figures and Tables

**Table 1:** Statistical distribution characteristics of rice milling process parameters and quality indicators for three rice varieties.

Indicator	Yangfunuo 4 (N = 177)	Qiuguang (N = 155)	Luhui 6684 (N = 168)
Processing parameters			
Milling time (s)	35.71 ± 19.65 [0, 60]	36.90 ± 20.28 [0, 60]	35.95 ± 19.77 [0, 60]
Milling speed (r/min)	823.73 ± 294.46 [0, 1050]	790.97 ± 319.77 [0, 1050]	854.46 ± 289.99 [0, 1050]
Physicochemical and nutritional attributes			
Amylose content (%)	1.48 ± 0.02 [1.44, 1.52]	16.58 ± 0.71 [15.45, 18.18]	23.14 ± 0.89 [21.90, 24.99]
Rapidly digestible starch (%)	40.17 ± 3.20 [36.10, 44.20]	27.88 ± 3.14 [21.60, 31.40]	13.93 ± 1.39 [11.00, 16.00]
Slowly digestible starch (%)	22.66 ± 1.61 [20.40, 25.90]	33.34 ± 2.19 [30.90, 38.30]	30.84 ± 1.39 [28.60, 33.80]
Resistant starch (%)	24.59 ± 2.42 [20.40, 27.80]	27.05 ± 2.92 [23.10, 30.70]	44.49 ± 2.37 [39.80, 48.40]
Sensory scores			
Aroma score (0–20)	15.60 ± 2.80 [10.0, 19.0]	14.50 ± 2.99 [10.0, 20.0]	15.40 ± 1.51 [13.0, 17.0]
Appearance score (0–20)	16.10 ± 2.18 [12.0, 20.0]	16.00 ± 2.40 [12.0, 18.0]	14.10 ± 1.37 [12.0, 16.0]
Palatability score (0–30)	24.10 ± 2.69 [21.0, 29.0]	21.80 ± 4.34 [14.0, 29.0]	16.00 ± 2.05 [13.0, 18.0]
Taste score (0–25)	21.10 ± 2.02 [18.0, 23.0]	20.60 ± 2.55 [17.0, 24.0]	16.70 ± 0.82 [16.0, 18.0]
Texture of cooled rice score (0–5)	4.20 ± 0.79 [3.0, 5.0]	3.30 ± 0.48 [3.0, 4.0]	2.80 ± 0.42 [2.0, 3.0]

Note: A milling time of 0 s combined with a milling speed of 0 r/min denotes the unmilled brown-rice control, which defines the lower boundary of the processing space; it does not *indicate* that raw or uncooked kernels were assessed. All samples, including the unmilled brown-rice control and lightly milled samples, were cooked according to the same GB/T 15682-2025 procedure and evaluated using the same sensory protocol.

**Table 2:** Reverse optimization results of different varieties under the dual objectives of low RDS content and high palatability.

Cultivar	Optimization Feasibility	Recommended Processing Parameters	Predicted Quality Attributes
Qiuguang (*japonica* rice)	Feasible	Milling time: 38.5 s Milling speed: 1020 r/min	RDS content: 21.6% Palatability score: 26.5
Yangfunuo 4 (glutinous rice)	Infeasible	Milling time: 18.0 s Milling speed: 900 r/min	RDS content: 36.1% Palatability score: 22.5
Luhui 6684 (*indica* rice)	Infeasible	Milling time: 25.0 s Milling speed: 750 r/min	RDS content: 13.5% Palatability score: 18.5

**Table 3:** Ablation comparison of the feasibility of reverse optimization results by the process-feasibility constraint module in different target scenarios.

Experimental Scenario	Optimization Objective Setting	Model Grouping	Inverse Process Parameter Output	Prediction Error	Engineering Feasibility Judgment
A: Maximum resistant starch retention	RS ≥ 48% Palatability ≥ 15	Unconstrained model	Milling time: 0.5 Milling speed: 7500	0.8%	Infeasible (speed exceeds limit)
		RiceMambaOpt	Milling time: 22.5 Milling speed: 750	3.4%	Feasible (gentle low-speed)
B: High-whiteness appearance	Appearance ≥ 18 RDS ≤ 30%	Unconstrained model	Milling time: −5 Milling speed: 1200	2.1%	Infeasible (negative time value)
		RiceMambaOpt	Milling time: 48 Milling speed: 980	7.6%	Feasible (prolonged intensive milling)
C: Slow digestibility combined with high palatability	RDS ≤ 15% Palatability ≥ 18	Unconstrained model	Milling time: 80 Milling speed: 150	0.9%	Infeasible (time exceeds limit)
		RiceMambaOpt	Milling time: 35 Milling speed: 780	4.2%	Feasible (balanced parameter setting)

## Data Availability

The datasets supporting the findings of this study are provided in the article. Further inquiries can be directed to the corresponding author.

## Appendix A

**Table A1:** Initial physical characteristics of the three rice cultivars before milling.

Initial Characteristic	Yangfunuo 4	Qiuguang	Luhui 6684
Moisture content (%)	12.4 ± 0.2	12.1 ± 0.3	12.6 ± 0.2
Thousand-kernel weight (g)	22.8 ± 0.4	24.6 ± 0.5	26.1 ± 0.6
Kernel length (mm)	5.62 ± 0.08	5.35 ± 0.07	6.74 ± 0.10
Kernel width (mm)	2.91 ± 0.05	2.94 ± 0.04	2.32 ± 0.04
Length-to-width ratio	1.93 ± 0.04	1.82 ± 0.03	2.91 ± 0.06
Single-kernel compression hardness (N)	45.8 ± 2.7	52.6 ± 3.1	68.4 ± 3.8
Amylose content (%)	1.58 ± 0.04	16.68 ± 0.31	23.10 ± 0.42

Note: Values are presented as mean ± standard deviation. Moisture content, thousand-kernel weight, and amylose content were measured in three independent batches; kernel dimensions and compression hardness were measured using 30 intact kernels per cultivar.

**Table A2:** Inter-panelist agreement and replicate repeatability for the five trained-panel sensory attributes.

Sensory Attribute	ICC(2,*k*)	95% Confidence Interval	Kendall’s *W*	Concordance *p*-Value	Replicate CV (%)
Aroma	0.84	0.76–0.90	0.66	<0.001	6.8
Appearance	0.89	0.83–0.93	0.72	<0.001	5.5
Palatability	0.91	0.86–0.95	0.76	<0.001	4.9
Taste	0.88	0.81–0.93	0.71	<0.001	5.7
Texture of cooled rice	0.83	0.74–0.89	0.64	<0.001	7.2

Note: ICC(2,k) denotes a two-way random-effects, absolute-agreement intraclass correlation coefficient for the mean score of the 10 trained panelists. Replicate CV represents the coefficient of variation across three independently cooked and evaluated replicates.

**Table A3:** Sample-level physical-plausibility screening of TabDDPM-generated candidates for the descriptive augmented dataset.

Sequential Screening Stage	Acceptance Criterion	Number Rejected at This Stage	Percentage of 6500 Candidates (%)
Equipment and categorical validity	Milling time: 0–60 s; milling speed: 0–1050 r/min; cultivar restricted to Yangfunuo 4, Qiuguang, or Luhui 6684	48	0.74
Measurement scale and starch composition	All composition values nonnegative; RDS + SDS + RS within 80–95%; aroma and appearance within 0–20; palatability within 0–30; taste within 0–25; cooled-rice texture within 0–5	76	1.17
Cultivar-conditioned physicochemical envelope	Each physicochemical variable within the same-cultivar real-training range expanded by 5% of its interquartile range	147	2.26
Local process–quality support	Each output within the range of the five nearest same-cultivar real training samples, expanded by two training-fold standard deviations	91	1.40
Distributional screening (CMD and JS divergence)	Per-variable Jensen–Shannon divergence and correlation-structure deviation relative to the real training data within the 0.05 acceptance band	1638	25.20
Total rejected	Failure of at least one sequential criterion	2000	30.77
Final retained set	Passed all four sample-level checks; CMD = 0.018; maximum variable-wise JS divergence = 0.037	4500	69.23

Note: Rejection counts are sequential and mutually exclusive. A candidate rejected at an earlier stage was not reconsidered at a later stage. The four sample-level plausibility checks were applied first and removed 362 of the 6500 candidates; the remaining 4862 candidates then entered the distributional screening. The dataset-level CMD and the maximum variable-wise JS divergence of the finally retained set were calculated after all screening had been completed.

**Table A4:** Architecture, training, and inverse-optimization settings used for computational reproducibility.

Component	Parameter	Setting
Data partition	Independent holdout	100 real samples, separated before preprocessing or augmentation
Data partition	Development set	400 real samples
Cross-validation	Fold composition	320 real training samples and 80 real validation samples per fold
Cross-validation	Augmentation rule	TabDDPM fitted only to the 320 real training samples in each fold
Final augmentation	Synthetic-to-real ratio	9:1; 3600 synthetic samples generated from 400 real development samples
Preprocessing	Continuous variables	Z-score statistics fitted only on the corresponding real training subset
Preprocessing	Cultivar variable	Learned categorical embedding with dimension 8
TabDDPM	Modeled variables	Cultivar, milling time, milling speed, and nine quality attributes
TabDDPM	Denoising network	Three hidden layers, 256 units per layer, SiLU activation, dropout = 0.10
TabDDPM	Time embedding	128 dimensions
TabDDPM	Diffusion process	1000 diffusion steps with a cosine noise schedule
TabDDPM	Optimizer	AdamW; learning rate = 2 × 10^−4^; weight decay = 1 × 10^−5^
TabDDPM	Training	Batch size = 128; maximum = 3000 epochs; patience = 200 epochs
Mamba backbone	Number of blocks	2
Mamba backbone	Model/state dimensions	Model dimension = 64; state dimension = 16
Mamba backbone	Internal settings	Expansion factor = 2; convolution width = 4; dropout = 0.10
MoE module	Experts and routing	Three experts; top-1 routing; capacity factor = 1.25
MoE module	Expert architecture	64–128–64 multilayer perceptron with SiLU activation
Output head	Outputs	Mean and log variance for each of nine attributes; 18 outputs in total
Forward-model loss	Objective	Heteroscedastic Gaussian negative log-likelihood + 0.01 × router load-balancing loss
Forward-model optimizer	AdamW settings	Initial learning rate = 1 × 10^−3^; weight decay = 1 × 10^−4^
Forward-model training	Schedule	Cosine decay to 1 × 10^−5^; batch size = 64; maximum = 500 epochs
Forward-model training	Regularization	Early-stopping patience = 40 epochs; gradient norm clipped at 1.0
Inverse optimization	Initialization	32 uniformly sampled starting points within equipment bounds
Inverse optimization	Adam settings	Learning rate = 0.05; maximum = 2000 iterations
Inverse optimization	Process-feasibility constraint	λ = 10.0; minimum cumulative milling exposure Nmin = 160 nominal spindle revolutions
Inverse optimization	Convergence criterion	Relative loss change < 10^−6^ for 50 consecutive iterations
Randomness control	Seeds	2024, 2025, 2026, 2027, and 2028
Computing environment	Software	Python 3.10.13; PyTorch 2.2.1; CUDA 12.1
Computing environment	Hardware	NVIDIA GeForce RTX 3090 GPU with 24 GB memory

**Table A5:** Matched comparison of RiceMambaOpt with conventional, tabular, and neural-network baselines.

Model	Selected Configuration	Mean *R*^2^ ± SD
Quadratic RSM	Linear, quadratic, and two-factor interaction terms; ridge regularization selected by validation	0.781 ± 0.045
SVR	RBF kernel; *C* = 100; *ε* = 0.10; kernel scale selected by validation	0.749 ± 0.043
Random forest	500 trees; maximum feature fraction and minimum leaf size selected by validation	0.805 ± 0.034
MLP	Hidden dimensions 64–128–64; dropout 0.10; AdamW optimizer	0.838 ± 0.026
GPR	Matérn-3/2 kernel with automatic relevance determination; noise variance optimized on training data	0.858 ± 0.030
XGBoost	800 trees; maximum depth 4; learning rate 0.03; subsample and feature fractions of 0.80	0.869 ± 0.025
CNN-LSTM	Two convolutional layers; LSTM hidden dimension 64; dropout 0.10	0.895 ± 0.016
CatBoost	800 trees; depth 6; learning rate 0.03; *L*_2_ regularization 5	0.904 ± 0.020
Transformer	Two encoder layers; four attention heads; model dimension 64; dropout 0.10	0.911 ± 0.016
RiceMambaOpt	Two Mamba blocks; model dimension 64; state dimension 16; three experts; top-1 routing	0.975 ± 0.008

**Table A6:** Component-ablation analysis of the Mamba and MoE modules.

Model Configuration	Mamba Module	Cultivar-Aware MoE	Mean *R*^2^ ± SD	Change Relative to RiceMambaOpt
Shared MLP	No	No	0.838 ± 0.026	−0.137
Mamba without MoE	Yes	No	0.949 ± 0.012	−0.026
MoE-MLP without Mamba	No	Yes	0.956 ± 0.011	−0.019
RiceMambaOpt	Yes	Yes	0.975 ± 0.008	Reference

**Table A7:** Target definitions, recommended milling conditions, experimental measurements, and profile-level errors of the prospective validation experiment.

Profile	Cultivar	Target Orientation	Target RDS (%)	Target Palatability Score	Recommended Milling Time (s)	Recommended Milling Speed (r/min)	Measured RDS (%), Mean ± SD	Measured Palatability Score, Mean ± SD	|Δ RDS| (Percentage Points)	|Δ Palatability| (Points)	Profile nRMSE (%)	All Prespecified Tolerances Met
YFN-1	Yangfunuo 4	Digestibility-oriented	≤33.0	≥20.0	12.0	900	36.6 ± 0.8	20.6 ± 0.4	3.6	0.6	9.3	No (measured RDS above tolerance)
YFN-2	Yangfunuo 4	Sensory-oriented	≤40.0	≥24.0	26.0	980	38.6 ± 0.9	24.9 ± 0.5	1.4	0.9	5.9	Yes
YFN-3	Yangfunuo 4	Balanced (I)	≤36.0	≥22.0	18.0	950	37.9 ± 0.7	22.7 ± 0.4	1.9	0.7	6.4	Yes
YFN-4	Yangfunuo 4	Balanced (II)	≤35.0	≥21.0	15.5	920	36.2 ± 0.8	22.1 ± 0.5	1.2	1.1	5.5	Yes
QG-1	Qiuguang	Digestibility-oriented	≤20.0	≥22.0	24.0	850	22.1 ± 0.6	22.5 ± 0.3	2.1	0.5	6.6	Yes
QG-2	Qiuguang	Sensory-oriented	≤22.0	≥26.0	38.5	1020	23.5 ± 0.7	26.8 ± 0.4	1.5	0.8	5.7	Yes
QG-3	Qiuguang	Balanced (I)	≤24.0	≥24.0	32.0	950	25.7 ± 0.6	25.0 ± 0.4	1.7	1.0	6.5	Yes
QG-4	Qiuguang	Balanced (II)	≤26.0	≥25.0	35.0	1000	27.3 ± 0.7	25.6 ± 0.3	1.3	0.6	5.4	Yes
LH-1	Luhui 6684	Digestibility-oriented	≤14.0	≥16.0	22.5	750	16.0 ± 0.5	16.9 ± 0.4	2.0	0.9	6.6	Yes
LH-2	Luhui 6684	Sensory-oriented	≤22.0	≥20.0	45.0	1000	20.4 ± 0.8	17.8 ± 0.6	1.6	2.2	9.2	No (measured palatability below tolerance)
LH-3	Luhui 6684	Balanced (I)	≤17.0	≥18.0	30.0	820	18.8 ± 0.6	18.7 ± 0.4	1.8	0.7	6.1	Yes
LH-4	Luhui 6684	Balanced (II)	≤18.0	≥18.5	33.0	860	19.5 ± 0.6	19.3 ± 0.4	1.5	0.8	5.8	Yes
Summary	All three cultivars	12 profiles; 36 samples	—	—	—	—	—	—	MAE = 1.8	MAE = 0.9	Joint nRMSE = 6.7	10 of 12 (83.3%)

**Table A8:** Hyperparameter search spaces and selected configurations for all models included in the comparative analysis.

Model	Hyperparameter Search Space	Selected Configuration Used for Final Refitting
Quadratic RSM	Full linear, interaction, and squared terms; ordinary least squares or ridge regularization with α ∈ {0, 10^−6^, 10^−4^, 10^−2^, 10^−1^}	Full second-order polynomial; ridge α = 10^−4^
SVR	Kernel ∈ {linear, RBF}; C ∈ {1, 10, 100, 1000}; γ ∈ {scale, 0.001, 0.01, 0.05, 0.1}; ε ∈ {0.01, 0.05, 0.10, 0.20}	RBF kernel; C = 100; γ = 0.05; ε = 0.05
RF	Trees ∈ {200, 500, 800, 1200}; maximum depth ∈ {None, 8, 12, 16, 24}; minimum samples per split ∈ {2, 4, 8}; minimum samples per leaf ∈ {1, 2, 4}; maximum features ∈ {sqrt, 0.5, 0.7, 1.0}	800 trees; maximum depth = 16; minimum samples per split = 2; minimum samples per leaf = 1; maximum features = 0.7
XGBoost	Trees ∈ {300, 500, 800, 1000}; maximum depth ∈ {3, 4, 6, 8}; learning rate ∈ {0.01, 0.03, 0.05, 0.10}; subsample ∈ {0.7, 0.8, 1.0}; column subsample ∈ {0.7, 0.8, 1.0}; minimum child weight ∈ {1, 3, 5}; L1 penalty ∈ {0, 10^−3^, 10^−2^}; L2 penalty ∈ {0.1, 1, 10}	1000 trees; maximum depth = 4; learning rate = 0.03; subsample = 0.8; column subsample = 0.8; minimum child weight = 1; L1 penalty = 0; L2 penalty = 1; early-stopping patience = 50 rounds
GPR	Kernel ∈ {RBF, Matérn-3/2, Matérn-5/2}; isotropic or automatic-relevance-determination length scale; noise α ∈ {10^−8^, 10^−6^, 10^−4^, 10^−2^}; optimizer restarts ∈ {5, 10, 20}	Automatic-relevance-determination RBF kernel + white-noise kernel; α = 10^−6^; 10 optimizer restarts
CatBoost	Iterations ∈ {300, 500, 800, 1000}; depth ∈ {4, 6, 8, 10}; learning rate ∈ {0.01, 0.03, 0.05, 0.10}; L2 leaf penalty ∈ {1, 3, 5, 10}; bagging temperature ∈ {0, 0.5, 1, 2}	1000 iterations; depth = 6; learning rate = 0.03; L2 leaf penalty = 3; bagging temperature = 1; RMSE loss; early-stopping patience = 50 rounds
MLP	Hidden layers ∈ {64–32, 128–64, 256–128–64}; dropout ∈ {0, 0.10, 0.20, 0.30}; learning rate ∈ {10^−4^, 5 × 10^−4^, 10^−3^}; weight decay ∈ {0, 10^−5^, 10^−4^, 10^−3^}; batch size ∈ {32, 64, 128}	Hidden layers = 128–64; SiLU activation; dropout = 0.20; AdamW; learning rate = 10^−3^; weight decay = 10^−4^; batch size = 64; maximum = 500 epochs; patience = 40 epochs
CNN–LSTM	Convolution filters ∈ {32, 64, 128}; kernel width ∈ {2, 3, 4}; LSTM units ∈ {32, 64, 128}; dense units ∈ {32, 64, 128}; dropout ∈ {0.10, 0.20, 0.30}; learning rate ∈ {10^−4^, 5 × 10^−4^, 10^−3^}; batch size ∈ {32, 64, 128}	64 convolution filters; kernel width = 3; LSTM units = 64; dense units = 64; dropout = 0.20; AdamW; learning rate = 5 × 10^−4^; weight decay = 10^−4^; batch size = 64; maximum = 500 epochs; patience = 40 epochs
Transformer	Model dimension ∈ {32, 64, 128}; attention heads ∈ {2, 4, 8}; encoder layers ∈ {1, 2, 3}; feed-forward dimension ∈ {64, 128, 256}; dropout ∈ {0.10, 0.20, 0.30}; learning rate ∈ {10^−4^, 5 × 10^−4^, 10^−3^}; batch size ∈ {32, 64, 128}	Model dimension = 64; 4 attention heads; 2 encoder layers; feed-forward dimension = 128; dropout = 0.10; AdamW; learning rate = 5 × 10^−4^; weight decay = 10^−4^; batch size = 64; maximum = 500 epochs; patience = 40 epochs
RiceMambaOpt	Mamba blocks ∈ {1, 2, 3}; model dimension ∈ {32, 64, 128}; state dimension ∈ {8, 16, 32}; expansion factor ∈ {1, 2, 4}; convolution width ∈ {2, 4, 8}; experts ∈ {2, 3, 4}; dropout ∈ {0.05, 0.10, 0.20}; learning rate ∈ {10^−4^, 5 × 10^−4^, 10^−3^}; batch size ∈ {32, 64, 128}	2 Mamba blocks; model dimension = 64; state dimension = 16; expansion factor = 2; convolution width = 4; three experts with top-1 routing; expert architecture = 64–128–64; dropout = 0.10; AdamW; learning rate = 10^−3^; weight decay = 10^−4^; batch size = 64; maximum = 500 epochs; patience = 40 epochs

**Table A9:** Indicator-specific coefficient of determination (*R*^2^, mean ± standard deviation) of the seven prediction models shown in Figure 5 across five real-observation validation folds.

Quality Indicator	SVR	RF	XGBoost	MLP	CNN-LSTM	Transformer	RiceMambaOpt
Amylose content	0.854 ± 0.026	0.882 ± 0.021	0.915 ± 0.016	0.893 ± 0.019	0.932 ± 0.011	0.945 ± 0.009	0.988 ± 0.004
RDS	0.762 ± 0.031	0.824 ± 0.025	0.889 ± 0.019	0.856 ± 0.022	0.901 ± 0.014	0.921 ± 0.011	0.982 ± 0.006
SDS	0.745 ± 0.034	0.805 ± 0.027	0.876 ± 0.021	0.841 ± 0.024	0.895 ± 0.015	0.910 ± 0.012	0.976 ± 0.007
RS	0.758 ± 0.038	0.810 ± 0.030	0.882 ± 0.023	0.842 ± 0.027	0.898 ± 0.017	0.915 ± 0.013	0.978 ± 0.008
Aroma	0.715 ± 0.041	0.772 ± 0.033	0.845 ± 0.026	0.812 ± 0.029	0.880 ± 0.019	0.895 ± 0.015	0.966 ± 0.009
Appearance	0.745 ± 0.036	0.801 ± 0.029	0.868 ± 0.022	0.835 ± 0.025	0.895 ± 0.016	0.912 ± 0.012	0.972 ± 0.006
Palatability	0.732 ± 0.044	0.795 ± 0.035	0.858 ± 0.028	0.831 ± 0.031	0.892 ± 0.021	0.908 ± 0.016	0.975 ± 0.008
Taste	0.728 ± 0.039	0.788 ± 0.032	0.852 ± 0.025	0.825 ± 0.028	0.888 ± 0.018	0.902 ± 0.014	0.970 ± 0.007
Texture of cooled rice	0.705 ± 0.045	0.765 ± 0.036	0.835 ± 0.029	0.805 ± 0.032	0.875 ± 0.022	0.888 ± 0.017	0.964 ± 0.010
Overall mean	0.749 ± 0.043	0.805 ± 0.034	0.869 ± 0.025	0.838 ± 0.026	0.895 ± 0.016	0.911 ± 0.016	0.975 ± 0.008

Note: Indicator-specific values are expressed as mean $R^{2}$ ± standard deviation across the five leakage-controlled validation folds (*n* = 5). The overall mean was calculated across the nine indicator-specific mean $R^{2}$ values, and its standard deviation represents the between-indicator variation. RiceMambaOpt was compared with the next-best baseline, Transformer, using a two-sided exact Wilcoxon signed-rank test based on the nine paired indicator-specific mean $R^{2}$ values. RiceMambaOpt achieved a mean paired improvement of 0.064 (V = 45, *p* = 0.0039, rank-biserial correlation = 1.00). p<0.01.

**Table A10:** Sensitivity of RiceMambaOpt performance to the synthetic-to-real training ratio under leakage-controlled five-fold validation.

Synthetic-to-Real Training Ratio	Real Training Observations per Fold	Synthetic Training Observations per Fold	Real Validation Observations per Fold	Mean *R*^2^ ± SD
0:1, real only	320	0	80	0.932 ± 0.015
1:1	320	320	80	0.955 ± 0.012
3:1	320	960	80	0.970 ± 0.009
9:1	320	2880	80	0.975 ± 0.008

**Table A11:** Per-variable predictive performance of RiceMambaOpt on the untouched 100-sample real holdout set.

Predicted Quality Variable	Unit or Score Range	*R* ^2^	RMSE	MAE	nRMSE (%)
Amylose content	%	0.987	0.96	0.61	4.08
Rapidly digestible starch (RDS)	%	0.982	1.38	1.04	4.16
Slowly digestible starch (SDS)	%	0.971	0.84	0.62	4.69
Resistant starch (RS)	%	0.976	1.26	0.93	4.50
Aroma	0–20	0.963	0.48	0.36	4.80
Appearance	0–20	0.969	0.35	0.27	4.38
Palatability	0–30	0.978	0.68	0.51	4.25
Taste	0–25	0.974	0.44	0.33	5.50
Texture of cooled rice	0–5	0.972	0.13	0.10	4.33
Mean	—	0.975	—	—	4.52

**Table A12:** Per-variable nRMSE (%) of all models on the untouched 100-sample real holdout set.

Model	Amylose	RDS	SDS	RS	Aroma	Appearance	Palatability	Taste	Texture of Cooled Rice	Mean
Quadratic RSM	10.80	15.20	16.50	15.90	17.80	17.00	18.20	17.30	17.60	16.26
SVR	9.80	16.50	17.80	17.20	18.50	17.90	19.10	18.70	18.20	17.08
RF	8.70	14.20	15.80	14.90	16.20	15.40	16.80	16.00	15.70	14.86
XGBoost	6.90	10.70	12.30	11.40	13.20	12.10	13.00	12.60	12.40	11.62
GPR	6.30	10.10	11.70	10.80	12.80	11.60	12.50	12.00	11.90	11.08
MLP	7.50	12.10	13.70	12.90	14.50	13.60	14.30	14.00	13.80	12.93
CNN-LSTM	6.10	9.40	10.80	10.00	11.50	10.70	11.40	11.00	10.90	10.20
CatBoost	5.80	9.00	10.10	9.60	10.80	10.00	10.50	10.20	10.00	9.56
Transformer	5.60	8.60	9.70	9.10	10.50	9.60	10.20	9.90	9.80	9.22
RiceMambaOpt	4.08	4.16	4.69	4.50	4.80	4.38	4.25	5.50	4.33	4.52

Note: RMSE and MAE retain the original unit of each variable. The nRMSE was normalized using the observed real-data range of the corresponding output variable. All values were calculated after model and hyperparameter selection using the same untouched real holdout samples.

**Table A13:** Uncertainty-calibration performance on the untouched 100-sample real holdout set.

Nominal Prediction-Interval Coverage	Empirical Coverage Probability	Absolute Coverage Error	Normalized Mean Prediction-Interval Width
80%	79.0%	1.0 percentage point	0.228
90%	88.4%	1.6 percentage points	0.286
95%	93.1%	1.9 percentage points	0.337
Mean	—	1.5 percentage points	—

**Table A14:** Quantitative comparison of representative RSM optimization studies and the matched internal comparison between quadratic RSM and RiceMambaOpt. (**A**) Matched internal comparison using identical data partitions, targets, and equipment bounds. (**B**) Representative published RSM optimization results and the present RiceMambaOpt solution.

**(A)**					
**Evaluation Item**		**Quadratic RSM**		**RiceMambaOpt**	
Mean forward-prediction R^2^		0.781 ± 0.045		0.975 ± 0.008	
Mean forward-prediction nRMSE (%)		16.26		4.52	
Holdout milling-time MAE (s)		5.840		0.960	
Holdout milling-time R^2^		0.812		0.986	
Holdout milling-speed MAE (r/min)		86.700		19.522	
Holdout milling-speed R^2^		0.624		0.851	
Qiuguang recommended milling time (s)		42.1		38.5	
Qiuguang recommended milling speed (r/min)		1050		1020	
Qiuguang predicted RDS (%)		24.8		21.6	
Qiuguang predicted palatability score		24.9		26.5	
**(B)**					
**Study and Method**	**Material or Operation**	**Optimization Variables**	**Reported Optimal Condition**	**Representative Quantitative Outcome**	**Comparability with the Present Study**
Meng et al. [46], RSM	Brown-rice milling in a vertical circulation mill	Milling weight, filling ratio, and milling duration	345 g, filling ratio 2.6, and 30 s	Head-rice yield 76.21%; whiteness index 50.57; specific energy consumption 33.69 kJ/kg	Directly relevant to rice milling, but responses, equipment, cultivars, and control variables differ
Li et al. [47], RSM	Rice drying before storage and processing	Air temperature, humidity, initial moisture, air velocity, and tempering ratio	43.14 °C, 48.00%, 23.80%, 0.70 m/s, and 3.55, respectively	Whole-rice rate 70.458%; resistant starch 195.26 μg/g; validation error 4.68%	Demonstrates multivariable rice-process optimization but addresses drying rather than milling
Khan et al. [48], RSM and machine learning	Tempering drying for milling and physicochemical quality	Drying temperature, tempering time, and initial moisture	53 °C, 60 min, and 17.5%, respectively	Head rice 33%; amylose 18.54%; alkali spreading value 6.88; gel consistency 97.40 mm	Shows that RSM remains competitive for low-dimensional rice-processing responses
Present study, RiceMambaOpt	Moderate milling of Qiuguang rice	Milling time and milling speed	38.5 s and 1020 r/min	Predicted in vitro RDS 21.6%; predicted palatability 26.5	Targets starch-digestibility and sensory attributes; numerical outcomes are not directly comparable with the published RSM studies

**Table A15:** Sensitivity of per-variable prediction performance to TabDDPM augmentation.

Predicted Variable	*R*^2^ Without Synthetic Augmentation	*R*^2^ with 9:1 Augmentation	Change in *R*^2^	nRMSE Without Augmentation (%)	nRMSE with 9:1 Augmentation (%)
Amylose content	0.965	0.987	+0.022	6.90	4.08
Rapidly digestible starch (RDS)	0.941	0.982	+0.041	8.20	4.16
Slowly digestible starch (SDS)	0.919	0.971	+0.052	9.40	4.69
Resistant starch (RS)	0.928	0.976	+0.048	8.80	4.50
Aroma	0.910	0.963	+0.053	10.10	4.80
Appearance	0.925	0.969	+0.044	9.20	4.38
Palatability	0.936	0.978	+0.042	8.70	4.25
Taste	0.924	0.974	+0.050	9.50	5.50
Texture of cooled rice	0.940	0.972	+0.032	7.90	4.33
Mean	0.932	0.975	+0.043	8.74	4.52

**Table A16:** Fidelity of the cultivar- and output-specific surrogate diagnostic models relative to the RiceMambaOpt inverse optimization pipeline.

Cultivar	Surrogate Output	Fidelity $R^{2}$	RMSE	MAE
Luhui 6684	Milling speed	0.962	18.7 r/min	14.6 r/min
Luhui 6684	Milling time	0.971	1.21 s	0.94 s
Yangfunuo 4	Milling speed	0.951	22.4 r/min	17.8 r/min
Yangfunuo 4	Milling time	0.968	1.34 s	1.05 s
Qiuguang	Milling speed	0.967	16.9 r/min	13.2 r/min
Qiuguang	Milling time	0.975	1.08 s	0.82 s
Overall mean	—	0.966	—	—

Note: Fidelity metrics were calculated using 2000 independent quality-vector queries per cultivar. The reference values were the milling-time and milling-speed recommendations returned by the complete RiceMambaOpt inverse optimization pipeline. RMSE and MAE are reported in the original process units.
